# Intelligent Polymer Electrolytes for Next‐Generation Zinc‐Based Energy Storage Devices

**DOI:** 10.1002/advs.78546

**Published:** 2026-10-31

**Authors:** Sanath Kumar, Jhansirani Kesavan, Muhammad Asim, Atul Verma, Yen‐Pei Fu

**Affiliations:** ^1^ Department of Materials Science and Engineering National Dong Hwa University Shou‐Feng Hualien Taiwan

**Keywords:** electrode–electrolyte interface engineering, intelligent polymer electrolytes, ion solvation regulation, practical validation, zinc‐based aqueous energy storage

## Abstract

Intelligent polymer electrolytes for zinc‐based energy‐storage devices have attracted considerable attention as multifunctional platforms capable of simultaneously regulating ion transport, water activity, interfacial chemistry, and mechanical stability. This review presents a comprehensive overview of intelligent polymer electrolytes for zinc‐ion batteries (ZIBs), zinc–air batteries (ZABs), and zinc‐ion hybrid capacitors (ZIHCs), encompassing hydrogels, organo‐hydrogels, composite polymer electrolytes, and solid‐state polymer systems. Particular attention is given to the molecular and structural strategies underlying ion‐solvation regulation, selective ion transport, dendrite suppression, corrosion inhibition, antifreezing behavior, self‐healing, adhesion, and mechanical adaptability. Emerging functionalities, including self‐healing, antifreezing, self‐adhesive, stretchable, and temperature‐responsive behavior, are highlighted in the context of improving operational durability under mechanical deformation and extreme temperatures. Bio‐derived polymer electrolytes and the growing use of artificial intelligence, machine learning, and computational simulations for electrolyte discovery and optimization are also discussed. Finally, key challenges associated with scalability, reproducibility, manufacturing, cost, sustainability, and practical full‐cell validation are critically assessed. Future research should prioritize multifunctional, adaptive, sustainable, and scalable polymer architectures that concurrently regulate ion transport, interfacial reactions, mechanical integrity, and environmental stability. Collectively, this review provides a framework for the rational design of intelligent polymer electrolytes toward safer, more durable, flexible, and commercially viable zinc‐based energy‐storage technologies.

## Graphical abstract

Intelligent polymer electrolytes are organized into a three‐level hierarchy that advances zinc‐based energy storage from passive ion conduction toward active, adaptive, and intelligent operation. Molecular regulation of Zn^+^ coordination, water activity, desolvation, ion flux, and interfaces underpins adaptive self‐healing, anti‐freezing, adhesion, and mechanical responses, while feedback and AI/ML enable future self‐adaptive ZIBs, ZABs, and ZIHCs.

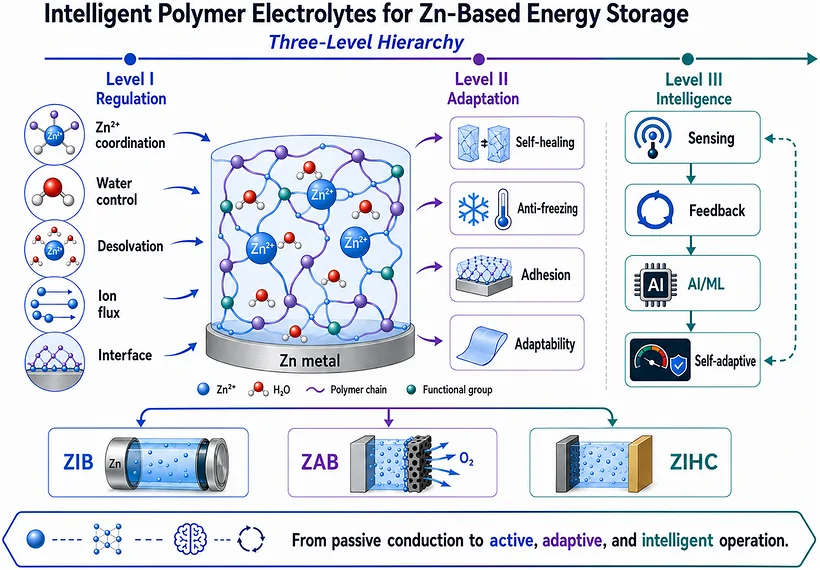

## 1 Introduction

The increasing deployment of renewable‐energy systems, portable electronics, wearable technologies, and electric transportation has intensified the demand for energy‐storage technologies that combine low cost, safety, and environmental sustainability. Zinc‐based energy‐storage devices, including zinc‐ion batteries (ZIBs), zinc‐air batteries (ZABs), and zinc‐ion hybrid capacitors (ZIHCs), have emerged as attractive alternatives to conventional lithium‐based technologies. Their appeal is underpinned by the abundance and low cost of zinc, together with its high theoretical capacity and favorable redox potential (−0.76 V vs. SHE) [1]. In addition, the widespread use of aqueous electrolytes substantially improves operational safety by avoiding the flammable organic solvents commonly used in conventional Li‐ion systems. These advantages make zinc‐based energy‐storage technologies promising candidates for applications ranging from portable electronics to large‐scale stationary storage. Despite these advantages, zinc‐based energy‐storage devices still face several interrelated challenges that limit practical deployment and long‐term reliability. At the Zn metal anode, repeated plating/stripping can produce spatially nonuniform deposition and progressive dendrite formation [2]. Dendritic growth can penetrate separators, induce internal short circuits, accelerate capacity decay, and compromise device safety. Aqueous Zn systems are also susceptible to parasitic interfacial reactions, particularly the hydrogen evolution reaction (HER), Zn corrosion, and the accumulation of passivating by‐products [3]. These processes consume active Zn and electrolyte, reduce Coulombic efficiency, increase interfacial resistance, and degrade electrochemical performance. Instability is further amplified by unfavorable Zn^2+^ solvation structures, heterogeneous ion flux, nonuniform electric‐field distributions, and inadequate interfacial protection [4]. Addressing these coupled phenomena is therefore essential for achieving highly reversible Zn electrochemistry. Polymer electrolytes provide a promising route because their interconnected networks can support efficient ion transport while offering mechanical constraint and chemical functionality that regulate Zn deposition. Functional groups such as carboxyl, sulfonic acid, and amino moieties can further tailor Zn^2+^ solvation, reduce water‐induced side reactions, decrease desolvation barriers, and stabilize the electrode–electrolyte interface, thereby improving reversibility and cycling durability [5]. Recent advances in polymer‐electrolyte design have substantially expanded the strategies available for stabilizing zinc‐based energy‐storage devices. Approaches including in situ polymerization, functional‐group engineering, bio‐inspired architectures, zwitterionic polymers, and composite polymer electrolytes have been developed to improve ionic conductivity, mechanical robustness, and interfacial stability [6]. Through these design routes, polymer electrolytes can homogenize Zn^2+^ flux, promote uniform Zn deposition, suppress dendritic growth, and mitigate parasitic reactions. More recently, stimuli‐responsive electrolytes that respond to temperature, pH, or mechanical damage have introduced adaptive ion‐transport and interface‐regulation functions, offering a pathway toward safer and more reliable operation under variable working conditions [7].

Although polymer, hydrogel, quasi‐solid‐state, and flexible electrolytes for zinc‐based batteries have been extensively reviewed, most previous reviews primarily organize the literature according to electrolyte composition, polymer class, device configuration, or individual functional strategies. Such classifications provide a useful overview of the field but do not fully address a central materials design question: how should polymer chemistry, Zn^2+^ coordination, ion transport, interfacial reactions, mechanical characteristics, and macroscopic electrochemical performance be quantitatively connected to establish transferable design principles for next‐generation zinc‐based polymer electrolytes. In particular, polymer electrolytes should not be evaluated solely according to ionic conductivity or cycling stability because these properties arise from strongly coupled molecular, structural, interfacial, and mechanical processes. The scientific challenge is therefore to identify how specific polymer structural features and electrolyte architectures regulate Zn^2+^ solvation and coordination, water activity, ion mobility, zinc deposition, parasitic reactions, mechanical deformation, and ultimately full‐cell performance.

This review critically examines recent progress in intelligent polymer electrolytes for zinc‐based energy‐storage devices. Rather than treating polymer electrolytes as a sequence of formulations, the discussion is organized around a mechanism‐to‐function‐to‐device framework: molecular interactions and network architecture govern ion and water regulation; these physicochemical processes determine interfacial and adaptive functions; and the resulting functions must ultimately be validated at the device level. The review first establishes the electrochemical characteristics of major Zn‐based systems and the limitations of conventional aqueous electrolytes, then analyzes ion‐transport mechanisms, functional‐group chemistry, polymer‐network structure, and electrode–electrolyte interface regulation. Major electrolyte classes, including hydrogels, organohydrogels, composite polymer electrolytes, and solid‐state polymer electrolytes, are compared with respect to their structural features, electrochemical functions, and inherent trade‐offs. Particular attention is given to self‐healing, antifreezing, self‐adhesive, and mechanically adaptive behavior, together with bio‐derived systems and the emerging use of artificial intelligence, machine learning, molecular dynamics, and density functional theory. A further distinguishing feature of this review is the integration of experimentally established polymer‐electrolyte chemistry with emerging computational and data‐driven approaches. Particular attention is given to the remaining gap between molecular‐level predictions and experimentally validated zinc‐battery performance. By combining molecular interactions, transport phenomena, interfacial chemistry, mechanical behavior, and computationally assisted materials discovery within a single analytical framework, this review aims to identify transferable design principles and unresolved questions for intelligent polymer electrolytes rather than providing another composition‐based catalog of reported electrolytes.

### 1.1 Major Zinc‐Based Systems and Their Mechanisms

The three most widely investigated zinc‐based energy‐storage technologies, ZABs, ZIBs, and ZIHCs, share a Zn metal anode but differ fundamentally in their cathodic reactions and charge‐storage mechanisms. In ZABs, reversible Zn oxidation/reduction at the anode is coupled to oxygen reduction (ORR) during discharge and oxygen evolution (OER) during charging at a porous air cathode. Because atmospheric O_2_ serves as the cathodic reactant, active cathode material does not need to be stored entirely within the cell, contributing to the high theoretical energy density of the system. ZAB performance is therefore strongly governed by the bifunctional catalytic activity of the air electrode and by the transport of OH^−^ and zincate species through the electrolyte, as illustrated in Figure 1a. In contrast, ZIBs generally involve reversible Zn plating/stripping at the anode together with Zn^2+^ insertion/extraction or related redox processes at the cathode. During discharge, Zn is oxidized, and Zn^2+^ migrates through the electrolyte toward host materials such as manganese oxides, vanadium oxides, Prussian blue analogs, or organic compounds; the reverse process occurs during charging. Accordingly, ZIB performance depends strongly on Zn^2+^ transport kinetics, cathode structural stability, and electrode–electrolyte interfacial reactions (Figure 1b). ZIHCs combine battery‐type Zn plating/stripping at the anode with capacitive ion adsorption and/or fast surface‐controlled pseudocapacitive reactions at a porous‐carbon or hybrid cathode. This hybrid mechanism enables high‐power capability and long cycle life while retaining a higher energy density than conventional electrochemical capacitors (Figure 1c). Understanding these mechanistic distinctions is essential for rational electrolyte and interface design. Representative cable‐type and sandwich‐type configurations for flexible Zn‐ based devices are shown in Figure 1d. Although ZIBs, ZABs, and ZIHCs share a zinc‐based negative electrode, their electrolyte requirements cannot be considered interchangeable. In mildly acidic ZIBs, electrolyte design primarily targets Zn^2+^ solvation, homogeneous ion flux, reversible electrodeposition, and compatibility with the cathode's ion‐storage mechanism. Alkaline ZABs require a different approach because hydroxide transport supports oxygen‐electrode reactions, while zincate formation and migration govern important aspects of zinc‐electrode chemistry. In ZIHCs, the electrolyte must simultaneously support reversible zinc plating/stripping and rapid ionic charge compensation at the capacitive or pseudocapacitive cathode. These differences influence not only the choice of polymer functional groups and solvent systems but also the appropriate methods for evaluating ion transport, interfacial stability, and full‐cell performance. Accordingly, the design principles discussed in this review should be interpreted in relation to the underlying electrochemical reactions rather than generalized across all zinc‐based devices.

**FIGURE 1:**
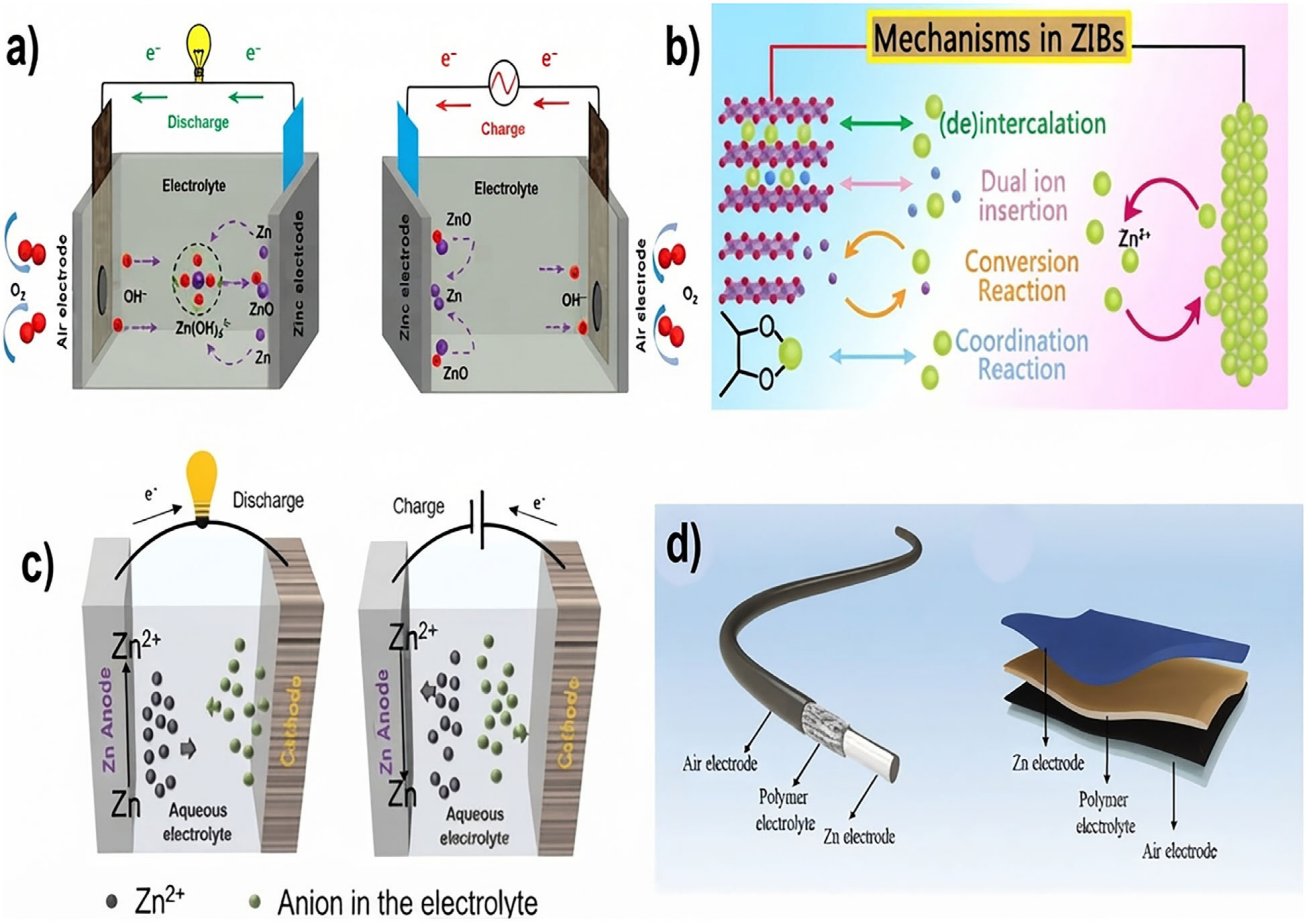
Representative mechanisms and device architectures for zinc‐based energy‐storage systems: (a) ZAB mechanism;reproduced with permission [8] 2023, RSC (b) ZIB mechanism;reproduced with permission [9] 2021, ELSEVIER (c) ZIHC mechanism;reproduced with permission [10] 2021, Wiley and (d) representative flexible device configurations:Reproduced with permission [8] 2023, RSC.

### 1.2 Challenges of Conventional Electrolytes

Electrolytes play a pivotal role in determining the electrochemical performance, safety, and operational stability of zinc‐based energy‐storage systems. Conventional aqueous electrolytes are generally safer than the flammable organic electrolytes used in Li‐ion batteries and have attracted considerable attention because of their high ionic conductivity, low cost, and intrinsic safety. Nevertheless, the presence of electrochemically active water introduces several interconnected degradation pathways that fundamentally limit long‐term device performance. As illustrated in Figure 2a,b, heterogeneous interfacial ion transport and electric‐field distribution promote nonuniform Zn nucleation and deposition, which progressively develops into dendritic growth. Simultaneously, direct contact between Zn and water induces parasitic hydrogen evolution and corrosion, while local changes in interfacial pH promote the precipitation of insulating basic zinc salts and other by‐products. These processes continuously consume active Zn and electrolyte, increase interfacial resistance, lower Coulombic efficiency, and may eventually lead to electrode passivation or internal failure. Figure 2c further highlights that electrolyte‐induced degradation is not restricted to the Zn anode; water‐related reactions and dissolution of active species at the cathode can further destabilize the electrode–electrolyte interface. In strongly alkaline systems, such as zinc‐air batteries, Zn corrosion, hydrogen evolution, passivation, and dendritic deposition can become particularly severe. Accumulated gas and uncontrolled side reactions may also cause battery swelling and introduce additional safety concerns. Beyond these interfacial issues, the temperature‐sensitive nature of aqueous electrolytes and their poor mechanical adaptability restrict their use under extreme environmental conditions and in flexible or wearable energy‐storage technologies as shown in Figure 2d. Therefore, overcoming the coupled limitations of water activity, Zn deposition instability, parasitic reactions, and interfacial degradation has become a central objective in developing next‐generation electrolytes for zinc‐based energy‐storage systems [11, 12].

**FIGURE 2:**
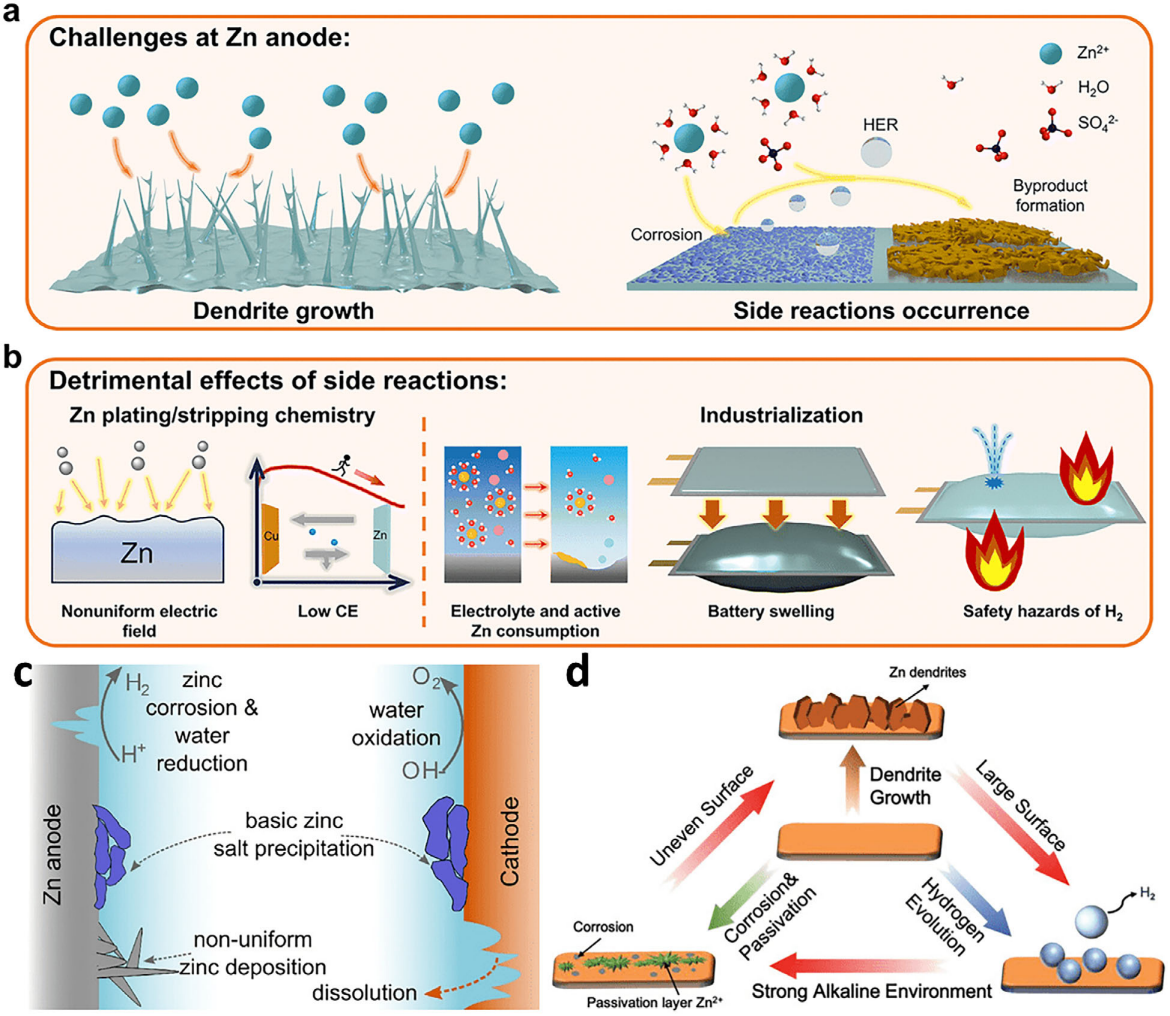
Major degradation mechanisms associated with conventional aqueous electrolytes in zinc‐based energy‐storage systems. (a) Zn dendrite growth and water‐induced side reactions; (b) consequences of interfacial side reactions [13]; (c) water‐induced side reactions [14]; (d) interrelated dendrite growth under strong alkaline conditions [15].

### 1.3 Emergence of Intelligent Polymer Electrolytes

The demand for safer, flexible, and higher‐performance zinc‐based energy‐storage devices has driven continuous innovation in electrolyte design. Conventional aqueous electrolytes offer high ionic conductivity and low manufacturing cost, but their susceptibility to side reactions, dendrite formation, leakage, and environmental instability restricts practical use. Gel polymer electrolytes were among the earliest approaches developed to address these limitations by immobilizing a liquid electrolyte within a polymer matrix, thereby reducing leakage while retaining much of the ionic conductivity of the aqueous phase. Building on this concept, hydrogel polymer electrolytes possess a combination of intrinsic physicochemical properties and functional capabilities that determine their suitability for zinc‐based energy‐storage systems. Intrinsic or passive characteristics, including ionic conductivity, water retention, and mechanical integrity, primarily establish the fundamental electrolyte environment for efficient ion transport and structural stability. In contrast, functional capabilities such as ion‐transport regulation, interfacial stabilization, self‐healing, and environmental responsiveness actively regulate electrochemical processes and enable the electrolyte to respond to changes occurring during battery operation. This transition reflects a broader shift in electrolyte design from passive ionic conduction toward active, multifunctional regulation of the electrochemical environment [16, 17]. Although a universally accepted definition is still emerging, intelligent polymer electrolytes can be broadly described as polymer‐based electrolyte systems that provide functions beyond simple ion conduction. In this review, the term “intelligent” is used for systems in which the polymer phase actively regulates operational variables such as ion coordination and transport, water activity, interfacial chemistry, mechanical damage, or environmental response rather than merely immobilizing a liquid electrolyte. Ionic conductivity represents a fundamental characteristic of hydrogel polymer electrolytes, governing the mobility of Zn^2+^ and other ionic species through the hydrated polymer network, which primarily reflects the intrinsic transport capability of the electrolyte rather than an actively responsive function. Water retention is another essential passive characteristic confined aqueous phase provides the medium for ionic dissociation and migration. Strong interactions between polymer functional groups and water molecules can suppress electrolyte dehydration, thereby maintaining ionic conductivity and preserving electrochemical operation during prolonged cycling. Furthermore, the polymer network also provides mechanical support, which helps maintain electrolyte integrity and dimensional stability during repeated electrochemical cycling. Crosslinking density, polymer–polymer interactions, and polymer‐solvent interactions collectively determine the mechanical robustness of the hydrogel without necessarily introducing an active electrochemical response. Beyond these passive characteristics, advanced hydrogel polymer electrolytes can incorporate functional capabilities that modify the electrode‐electrolyte environment through intrinsic or stimulus‐responsive physicochemical mechanisms. Such interactions can reconstruct local solvation structures, reorganize hydrogen‐bond networks, alter desolvation and charge‐transfer kinetics, and stabilize electrode interfaces. The defining advantage of intelligent polymer electrolytes is therefore their ability to couple electrochemical regulation with stimulus‐responsive mechanical or environmental adaptation within a single electrolyte platform [18, 19, 20].

A further hallmark of intelligent polymer electrolytes is the combination of multiple adaptive functionalities such as self‐healing, antifreezing, self‐adhesive, and stretchable behavior within a single material platform, and the introduction of an adaptive functionality that enables the polymer network to recover from mechanically induced defects during operation. Reversible hydrogen bonding, metal–ligand coordination, ionic interactions, or dynamic covalent bonds can reconstruct damaged network structures, thereby maintaining electrolyte continuity and interfacial contact after deformation or localized damage. Increasing attention is also being directed toward interface‐regulating electrolytes that control Zn^2+^ transport, stabilize electrode surfaces, and suppress dendritic growth through tailored polymer–ion and polymer–surface interactions. Consequently, modern polymer electrolytes are expected to provide not only ionic conduction but also interfacial regulation, mechanical resilience, environmental tolerance, and long‐term operational stability. Also, Environmental responsiveness enables certain polymer electrolytes to exhibit stimulus‐dependent adaptation from conventional passive polymer matrices. Changes in temperature, humidity, mechanical stress, or local chemical conditions can alter polymer‐chain interactions, water confinement, and ion mobility. Appropriately designed responsive networks can therefore adapt their physicochemical characteristics to changing operating conditions, supporting stable electrochemical performance across variable environments. This multifunctional design philosophy positions intelligent polymer electrolytes as a promising route for overcoming the coupled limitations of conventional electrolyte systems. The following sections therefore examine their fundamental design principles, structural classifications, and functional mechanisms, with emphasis on hydrogel, organohydrogel, composite, and solid‐state polymer electrolytes for ZIBs, ZABs, and ZIHCs. Emerging directions, including AI‐assisted electrolyte design, commercialization challenges, and future research opportunities, are also considered [21, 22]. For clarity, the functional capabilities of polymer electrolytes are discussed using a three‐level working framework that distinguishes passive physicochemical properties, stimulus‐responsive adaptive functionalities, and integrated sensing–feedback‐based intelligence. In this framework, Level II describes experimentally demonstrated responses to external or internal stimuli, whereas Level III is reserved for systems that integrate sensing, decision‐making and feedback‐controlled regulation. Functionalities that lack experimental evidence of closed‐loop operation are considered prospective Level III concepts rather than demonstrated intelligent behavior.

## 2 Fundamental Characteristics and Classification of Polymer Electrolytes

### 2.1 Chemical Composition and Molecular Interactions of Polymer Electrolytes

In zinc‐based energy storage systems, polymer electrolytes provide an ion‐conducting medium through a combination of polymer chain dynamics, ion hopping, and vehicular motion of solvated ions. In a polymer electrolyte, the zinc salt or alkaline electrolyte dissociates into mobile ions that interact with polar functional groups such as –OH, –COOH, –C = O, –O–, or ether groups present along the polymer backbone. For ZIBs and ZIHCs, Zn^2+^ ions migrate from the zinc anode toward the cathode during discharge, while the reverse transport occurs during charging; this migration is facilitated by repeated coordination/de‐coordination of Zn^2+^ with polymer functional groups and segmental motion of the polymer chains, which transiently opens free‐volume pathways for ion diffusion. Depending on the polymer matrix and solvent/plasticizer content, ion transport may also involve the movement of solvated Zn^2+^ complexes, whereas hydrogen bonding and ion–dipole interactions regulate ion mobility and transference number. In ZABs, OH^−^ is generally the principal charge‐carrying species in alkaline electrolytes, while soluble zinc‐containing species, predominantly Zn(OH)_4_^2−^ under alkaline conditions, participate in zinc‐side transport, deposition, and dissolution. The relative contribution of these species depends on electrolyte composition, concentration, and electrode operating conditions. Therefore, transport or transference numbers reported for ZAB electrolytes should be interpreted according to the specific definition and measurement method used in each original study rather than being directly equated with the conventional Zn^2+^ transference number used in zinc‐salt electrolytes. During operation, polymer‐chain flexibility, water content, salt concentration, free volume, and degree of crosslinking collectively determine ionic conductivity and ion diffusion kinetics. At the electrode–electrolyte interface, ions must undergo desolvation or partial de‐coordination before charge‐transfer reactions, making interfacial ion transport equally important to bulk diffusion. Polymer electrolytes can therefore establish a stable and confined ion‐transport pathway while suppressing electrolyte leakage, dendrite‐induced short circuits, parasitic reactions, and carbonation in alkaline ZAB electrolytes. In ZIHCs, the polymer electrolyte additionally supports rapid Zn^2+^ transport toward the battery‐type zinc electrode while maintaining ionic accessibility to the capacitive host, enabling coupled faradaic and surface‐controlled charge storage. Consequently, optimizing polymer polarity, segmental mobility, crosslinking density, solvent retention, and ion–polymer interactions is essential for achieving high ionic conductivity, efficient Zn^2+^/OH^−^ transport, low interfacial resistance, and stable operation over a broad temperature range.

### 2.2 Network Architecture and Structural Characteristics of Polymer Electrolytes

Polymer electrolytes have attracted significant attention in the development of safer, more efficient, and durable zinc‐based batteries. An ideal polymer electrolyte should maintain intimate contact with electrode surfaces, accommodate volume changes during cycling, minimize interfacial resistance, and provide sufficient mechanical and thermal stability for long‐term operation. These properties are largely governed by hydrophilic functional groups, particularly carboxyl (–COOH), hydroxyl (–OH), amide (–CONH_2_), and sulfonic (–SO_3_H), which regulate Zn^2+^ transport, water activity, and interfacial stability.

Carboxyl groups are commonly present in polyacrylic acid (PAA), carboxymethyl cellulose (CMC), and pectin. Upon ionization, they generate carboxylate (‐COO^−^) sites that strongly coordinate with Zn^2+^ through oxygen atoms, enabling efficient ion migration within the polymer network. Carboxyl groups also form hydrogen bonds with water molecules, modifying the Zn^2+^ hydration environment and reducing hydrogen evolution, zinc corrosion, and dendrite growth. In addition, Zn^2+^ ions act as reversible bridges between polymer chains, improving structural integrity, flexibility, and self‐healing behavior while maintaining ionic conductivity. Although PAA‐based electrolytes generally exhibit limited mechanical strength, reinforcement using aluminum oxide (Al_2_O_3_) nanoparticles has significantly enhanced their mechanical robustness and electrochemical performance, demonstrating an effective strategy for overcoming this limitation [23]. Hydroxyl groups, commonly found in PVA, cellulose, and chitosan derivatives, play an important role in hydrogel electrolytes because they readily form extensive hydrogen‐bonding networks. These interactions immobilize water molecules, suppress parasitic reactions at the zinc anode, and improve electrolyte stability. Hydroxyl oxygen atoms also weakly coordinate with Zn^2+^, lowering the desolvation energy barrier and facilitating rapid ion transfer at the electrode interface. Furthermore, hydroxyl groups reduce polymer chain packing, increase the amorphous character of the electrolyte, and create larger free volumes that promote Zn^2+^ migration while improving water retention [24]. Incorporation of silicon dioxide (SiO_2_) nanoparticles and carbon nanotubes (CNTs) into PVA‐based electrolytes has further improved water uptake and ionic conductivity. However, strong intermolecular hydrogen bonding in pure PVA often leads to excessive crystallinity, making the material stiff and brittle. Structural modification through electron beam irradiation has been shown to reduce crystallinity and markedly improve flexibility and elongation without requiring additional chemical crosslinkers [25]. Amide groups are present in polymers such as PAM, PVP, gelatin, and poly(N‐isopropylacrylamide) (PNIPAM). These polymers contain both hydrogen‐bond donor and acceptor sites, allowing the formation of robust hydrogen‐bonding networks that enhance mechanical stability, water retention, and ionic transport. Amide‐containing polymer electrolytes also exhibit excellent antifreezing characteristics, enabling battery operation over a broad temperature range. Among them, PNIPAM is particularly notable because of its thermo‐responsive behavior. Its molecular structure reversibly changes with temperature, regulating Zn^2+^ transport through a switchable ion‐gating mechanism. Incorporation of acrylamide (AM) and 1‐vinylimidazole into PNIPAM further improves this temperature‐responsive regulation, allowing efficient ion transport below the lower critical solution temperature while restricting Zn^2+^ migration at higher temperatures to improve thermal safety. Despite these advantages, some amide‐based polymers, particularly PAM and gelatin, possess relatively low structural toughness, making them susceptible to mechanical damage and dendrite penetration during prolonged battery operation [26]. Sulfonic groups are incorporated into polymers such as poly(styrene sulfonate) (PSS), sulfonated poly(ether ether ketone) (SPEEK), and (poly(3,4‐ethylenedioxythiophene) polystyrene sulfonate) (PEDOT:PSS). These polymers dissociate readily in aqueous electrolytes, generating fixed sulfonate groups that create continuous ion‐conduction pathways for Zn^2+^ while restricting the movement of anions. As a result, sulfonic polymers increase the Zn^2+^ transference number, reduce concentration polarization, and enhance ionic conductivity. Compared with carboxyl‐ and hydroxyl‐based polymers, sulfonic acid‐functionalized polymers generally exhibit higher intrinsic ionic conductivity because of their stronger acidity and greater degree of ionization. In PEDOT:PSS, the PSS component provides ionic conduction, while PEDOT contributes electronic conductivity, making these materials effective as conductive interfacial layers that simultaneously regulate ion and electron transport. Sulfonate‐functionalized chitosan has also demonstrated improved ionic conductivity, flexibility, and uniform zinc deposition, highlighting the effectiveness of sulfonic groups in enhancing both electrochemical and mechanical properties.

Overall, the incorporation of functional groups enables polymer electrolytes to regulate Zn^2+^ transport, control water activity, suppress dendrite formation, reduce side reactions, and improve mechanical stability. By combining these functionalities within tailored polymer networks, next‐generation polymer electrolytes can simultaneously achieve high ionic conductivity, strong interfacial compatibility, excellent structural durability, and stable long‐term electrochemical performance, making them promising candidates for advanced zinc‐based energy storage systems. The distinct coordination chemistry, electrolyte functions, and design trade‐offs associated with the principal polymer functional groups are summarized in Table 1.

**TABLE 1:** Functional‐group‐level design roles in intelligent polymer electrolytes for zinc‐based energy storage.

Polymer class	Key interactions	Ion‐transport mechanism	Main functions	Key limitation
Carboxyl/carboxylate PAA, CMC, pectin, SA	Zn^2+^–O coordination H‐bonding Electrostatic interactions	Reversible Zn^2+^ coordination/decoordination Hydrated pathways	Zn^2+^ flux regulation Uniform deposition HER/dendrite suppression Adhesion/self‐healing	Strong Zn^2+^ binding may reduce mobility
Hydroxyl PVA, cellulose, chitosan	H‐bonding Weak Zn^2+^ coordination	Hydrated‐ion transport Segmental motion	Water regulation Zn^2+^ transport Antifreezing Desolvation control	Strong H‐bonding may increase crystallinity
Amide PAM, PVP, gelatin, PNIPAM	Zn^2+^–carbonyl/amide coordination H‐bonding	Coordination‐assisted Zn^2+^ migration Segmental transport	Zn^2+^ solvation regulation Water retention Interfacial stabilization Thermal responsiveness	Dense networks may restrict ion transport
Fixed‐anion / polyanionic PSS, SPEEK, AMPS	Fixed –SO_3_^−^ groups Electrostatic interactions	Cation‐selective transport Hydrated ionic channels Restricted anion motion	Increased cation selectivity Zn^2+^ flux homogenization Reduced concentration polarization	Swelling/ionic association may reduce stability or mobility
Zwitterionic SBMA, DMAPS	Zwitterionic electrostatics Ion–dipole interactions Strong hydration	Hydrated ionic pathways Zn^2+^ interaction with anionic sites	Zn^2+^ solvation/desolvation control Uniform deposition Adhesion Antifreezing	Strong ion–polymer interaction may immobilize ions
PEDOT:PSS Conducting polymer–polyelectrolyte composite	PEDOT:PSS interaction Ionic/H‐bond interactions	Dual ion/electron transport PSS: ionic conduction PEDOT: electronic conduction	Ionic/electronic conductivity Interfacial charge transfer Electrode‐interface regulation	Ion/electron transport balance required

### 2.3 Physicochemical Properties and Functional Roles of Hydrogel Electrolytes

The electrode–electrolyte interface is the most critical region in zinc‐based energy storage systems, as it governs Zn^2+^ desolvation, charge transfer, zinc deposition/stripping, and cathode redox reactions. Consequently, its stability directly determines Coulombic efficiency, reaction kinetics, cycling stability, and overall battery performance. During repeated charge–discharge cycles, this interface continuously evolves owing to changes in local ion concentration, surface chemistry, and electrode morphology. Such dynamic changes often trigger hydrogen evolution, zinc corrosion, dendrite formation, by‐product accumulation, and active‐material dissolution, ultimately leading to performance degradation. Therefore, stabilizing the electrode–electrolyte interface has become one of the fundamental design principles of intelligent polymer electrolytes [27]. Unlike conventional aqueous electrolytes, intelligent polymer electrolytes actively regulate the electrode–electrolyte interface by controlling interactions among polymer functional groups, Zn^2+^ ions, and water molecules. Through rational molecular design, polymer matrices modify the Zn^2+^ solvation structure, reduce water activity, and facilitate Zn^2+^ desolvation before electrochemical deposition. These effects improve ion transport, suppress undesirable side reactions, and promote uniform zinc deposition, thereby enhancing the stability and reversibility of zinc‐based batteries. Various polymer systems have demonstrated these interfacial advantages. Poly(ethylene glycol) diacrylate (PEGDA), polyacrylamide (PAM), and carboxymethyl cellulose (CMC) form a hydrogel electrolyte in which the amide groups of PAM coordinate with Zn^2+^, thereby regulating its local coordination environment, as shown in Figure 3a [28]. Such polymer‐ion interactions facilitate the removal of coordinated water molecules and reduce the energetic barrier for Zn^2+^ desolvation, as supported by the calculated desolvation energies shown in Figure 3b. Consequently, Zn^2+^ transfer across the interface is facilitated, while reduced water participation suppresses parasitic reactions and promotes more homogeneous Zn deposition [163]. Similarly, sulfobetaine‐containing polymer electrolytes regulate the local coordination environment of Zn^2+^ by partially replacing coordinated water molecules through polymer‐ion interactions. The reconstructed solvation structure lowers the desolvation barrier, suppresses hydrogen evolution by reducing water reactivity, and accelerates interfacial charge‐transfer kinetics.

**FIGURE 3:**
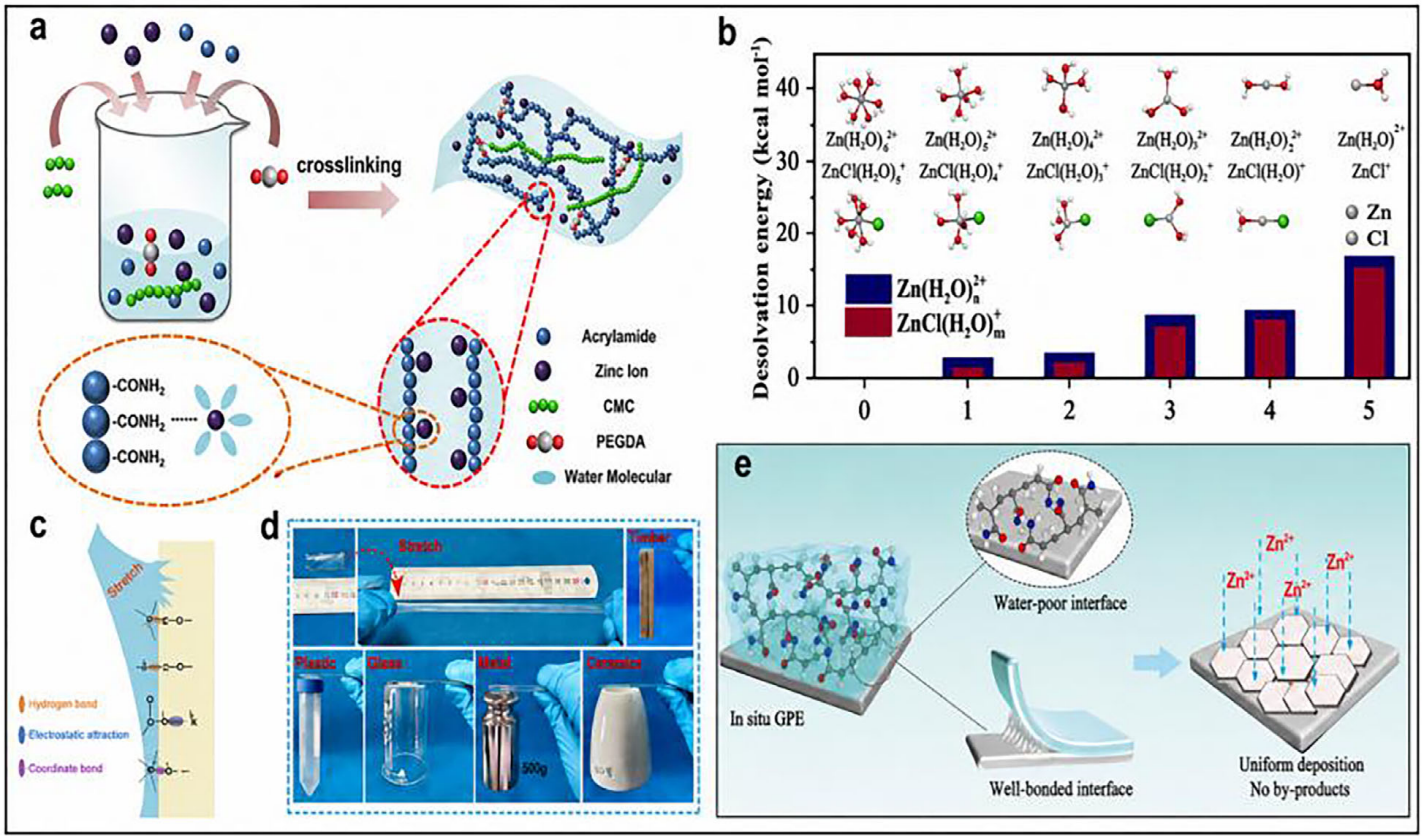
Interfacial regulation by intelligent polymer electrolytes. (a) Polymer‐network formation and Zn^2+^coordination; reproduced with permission [28] 2026, Elsevier, (b) regulation of Zn^2+^ desolvation energy, reproduced with permission [163] 2025, Wiley (c, d) interfacial bonding and adhesive/mechanical adaptability;reproduced with permission [30],2026, wiley and (e) formation of a water‐poor, well‐bonded interface enabling homogeneous Zn^2+^ transport, uniform Zn deposition, and suppressed by‐product formation; reproduced with permission [29], 2022, Wiley.

Polyvinyl alcohol (PVA)‐based electrolytes further demonstrate the importance of water regulation by forming extensive hydrogen‐bonding networks with water molecules. These interactions reduce water activity, facilitate Zn^2+^ transport, and stabilize both Zn anodes and cathode materials by minimizing active‐material dissolution and irreversible by‐product formation. More importantly, constructing a polymer electrolyte directly at the electrode surface can generate a water‐poor and well‐bonded interface, as schematically illustrated in Figure 3e. Such an interface limits direct contact between active water and Zn while homogenizing interfacial Zn^2+^ flux, thereby promoting uniform Zn deposition and suppressing dendrites and interfacial by‐products [29]. Interfacial stability is also strongly influenced by the physical contact between the electrolyte and electrodes, particularly in flexible energy‐storage systems. Hydrogels composed of acrylamide (AM), 3‐(methacryloylamino)propyltrimethylammonium chloride (MAPTAC), and gelatin combine efficient ion conduction with strong interfacial adhesion. As illustrated in Figure 3c, hydrogen bonding, electrostatic attraction, and coordination bonding establish multiple reversible interactions with contacting surfaces. These interactions enable the hydrogel to sustain substantial deformation and adhere strongly to chemically diverse substrates, including plastics, glass, metals, ceramics, and wood (Figure 3d). Such adhesive characteristics help preserve intimate electrode–electrolyte contact during mechanical deformation and minimize interfacial separation. Meanwhile, the cationic polymer framework provides continuous ion‐conduction pathways that homogenize Zn^2+^ flux, promote preferential deposition on stable crystal planes, and suppress dendritic growth [30].

Overall, intelligent polymer electrolytes improve battery performance through multiple synergistic mechanisms, including Zn^2+^ solvation regulation, water activity control, uniform ion transport, interfacial adhesion, and stabilization of electrode surfaces. Rather than functioning solely as ion‐conducting media, these materials actively regulate interfacial electrochemical processes, leading to enhanced cycling stability, reduced parasitic reactions, suppressed dendrite formation, and improved long‐term durability. Consequently, interface engineering using advanced polymer electrolytes has become a key strategy for developing high‐performance and reliable zinc‐based energy storage systems.

### 2.4 Contradictory Structure–Property Relationships and Their Possible Origins

Although hydrogel electrolytes have demonstrated significant potential for zinc‐based energy storage, reported structure‐property relationships are not always consistent across different studies. Such apparent contradictions often originate from the strongly coupled effects of polymer chemistry, network architecture, electrolyte composition, water activity, and testing conditions rather than from fundamentally incompatible mechanisms. For example, increasing the crosslinking density can enhance mechanical strength, dimensional stability, and resistance to deformation, thereby promoting more uniform Zn deposition; however, excessive crosslinking can restrict polymer‐chain mobility and free volume, resulting in reduced Zn^2+^ diffusion and ionic conductivity. Similarly, stronger coordination between Zn^2+^ and functional groups such as carboxyl, hydroxyl, amide, ether, or sulfonate groups may regulate Zn^2+^ flux and modify the Zn^2+^ solvation environment, but excessively strong interactions can immobilize Zn^2+^ and increase the energetic barrier for ion migration. Water content represents another important source of competing effects. Increasing water content generally facilitates salt dissociation and ion transport, but excessive free water can accelerate hydrogen evolution, zinc corrosion, and other parasitic reactions. Consequently, the condition producing maximum ionic conductivity does not necessarily provide optimum cycling stability. Salt concentration can exhibit a similar non‐monotonic relationship: increasing Zn‐salt concentration may initially increase charge‐carrier concentration, whereas excessive concentration can promote ion pairing, aggregation, and increased viscosity, thereby reducing effective Zn^2+^ mobility. Importantly, comparisons among reported systems are further complicated by differences in polymer concentration, hydrogel thickness, temperature, Zn‐electrode thickness, electrolyte loading, current density, areal capacity, and depth of discharge. Therefore, ionic conductivity alone cannot adequately describe Zn^2+^ transport or predict zinc‐battery performance; Zn^2+^ transference number, diffusion coefficient, activation energy, mechanical properties, interfacial resistance, and Zn plating/stripping behavior should be considered simultaneously. Within the hierarchical structure‐property‐interface‐performance framework, these apparently conflicting observations can therefore be reconciled as competing contributions of molecular interactions, network structure, ion transport, and interfacial processes. The objective of hydrogel‐electrolyte design should consequently be to identify an optimum balance among these coupled parameters rather than maximizing any single property independently.

The structure, property, interface, and performance framework established above provides a basis for understanding how polymer chemistry and network architecture influence Zn^2+^ coordination, ion transport, interfacial reactions, and macroscopic battery performance. However, polymer electrolytes encompass a chemically and structurally diverse family of materials, and these systems cannot be meaningfully compared by treating them as a single homogeneous category. Therefore, a rational classification is necessary to organize this diverse literature and, more importantly, to identify the structure–property relationships that are common across different polymer‐electrolyte platforms. In the following section, the polymer electrolytes are consequently classified according to their dominant structural and physicochemical characteristics rather than solely according to application or device configuration. This organization enables comparison of different electrolyte systems using common descriptors, including Zn^2+^ coordination, ionic conductivity, Zn^2+^ transference, mechanical integrity, water regulation, and interfacial stability. Such a classification also provides a logical basis for subsequently examining how specific polymer architectures and chemical functionalities can be selected or combined to address the coupled requirements of zinc‐based energy‐storage systems.

## 3 Classification of Intelligent Polymer Electrolytes

The polymer electrolytes discussed in this section are classified according to their dominant structural and solvent‐state characteristics, including hydrogels, organo‐hydrogels, composite polymer electrolytes, and solid‐state polymer electrolytes. Within each class, it is important to distinguish the intrinsic or passive characteristics of the polymer matrix from deliberately engineered functional capabilities. Ionic conductivity, water retention, and mechanical integrity primarily define the fundamental physicochemical properties of an electrolyte, whereas ion‐transport regulation, interfacial stabilization, self‐healing, and environmental responsiveness represent active functionalities introduced through molecular interactions, network architecture, additives, or stimuli‐responsive components. Accordingly, the following sections examine not only the composition and structure of each polymer platform but also how specific design modifications transform inherent electrolyte properties into functional regulation of Zn^2+^ transport, water activity, electrode interfaces, mechanical damage, and environmental conditions.

### 3.1 Hydrogel Polymer Electrolytes

Hydrogels are three‐dimensional crosslinked polymer networks capable of absorbing and retaining large quantities of water while preserving macroscopic structural integrity. Their high solvent content, ionic conductivity, water retention, and mechanical flexibility constitute the fundamental physicochemical characteristics that enable their use as quasi‐solid electrolytes. The hydrated polymer network provides continuous pathways for ion migration, while the crosslinked framework immobilizes the liquid phase and maintains structural integrity during deformation. These characteristics, however, do not by themselves constitute intelligent behavior. Functional capabilities emerge when polymer chemistry and network architecture are deliberately designed to regulate Zn^2+^ transport, modify solvation and water activity, stabilize electrode–electrolyte interfaces, or respond dynamically to mechanical and environmental stimuli. Accordingly, recent hydrogel research has progressed from optimizing inherent conductivity and mechanical properties toward active regulation of ion flux, interfacial reactions, self‐healing, antifreezing behavior, and temperature adaptability. The following sections therefore discuss representative PVA‐, PAM‐, PAA‐, and sodium alginate‐based hydrogels by distinguishing their intrinsic electrolyte properties from the functional mechanisms introduced through molecular and structural engineering.

#### 3.1.1 PVA‐Based Hydrogels

PVA is recognized as a leading polymer matrix for hydrogel electrolytes due to its excellent film‐forming capability, hydrophilicity, affordability, non‐toxicity, and rich hydroxyl group content. PVA chains can be crosslinked chemically using various agents or physically through freeze‐thaw treatments, resulting in a robust three‐dimensional porous network. The crosslinked network provides electrolyte retention and mechanical integrity, while its pore structure and crosslink density influence ionic transport and flexibility. The structural attributes of PVA hydrogels make them particularly attractive for flexible zinc‐based energy storage devices [31]. Recent advances in PVA‐based hydrogel electrolytes have demonstrated that introducing functional biomolecules can transform passive polymer matrices into active ion‐transport media. Xu et al. incorporated α‐helical protein molecules into a PVA network to construct a superelastic hydrogel electrolyte with interconnected Zn^2+^ transport pathways. As illustrated in Figure 4a, the hierarchical α‐helical protein structure provides abundant polar coordination sites while preserving continuous pathways for rapid Zn^2+^ migration. The resulting α‐HP/PVA hydrogel exhibited an ionic conductivity of 93.8 mS cm^−^^1^, substantially higher than pristine PVA. The abundant amide groups within the protein competitively coordinated with Zn^2+^ through nitrogen and oxygen atoms, reducing the migration energy barrier from 0.56 to 0.37 eV and thereby facilitating rapid ion transport. The combined effects of protein‐mediated Zn^2+^ coordination and network reinforcement improved ion transport and interfacial stability, contributing to cycling stability exceeding 5300 h in Zn‖Zn symmetric cells. The synergistic regulation of ion transport, solvation structure, and electrode interface resulted in remarkable cycling stability exceeding 5300 h in Zn||Zn symmetric cells, highlighting the potential of biomolecule‐assisted PVA hydrogels for next‐generation zinc metal batteries [32].

**FIGURE 4:**
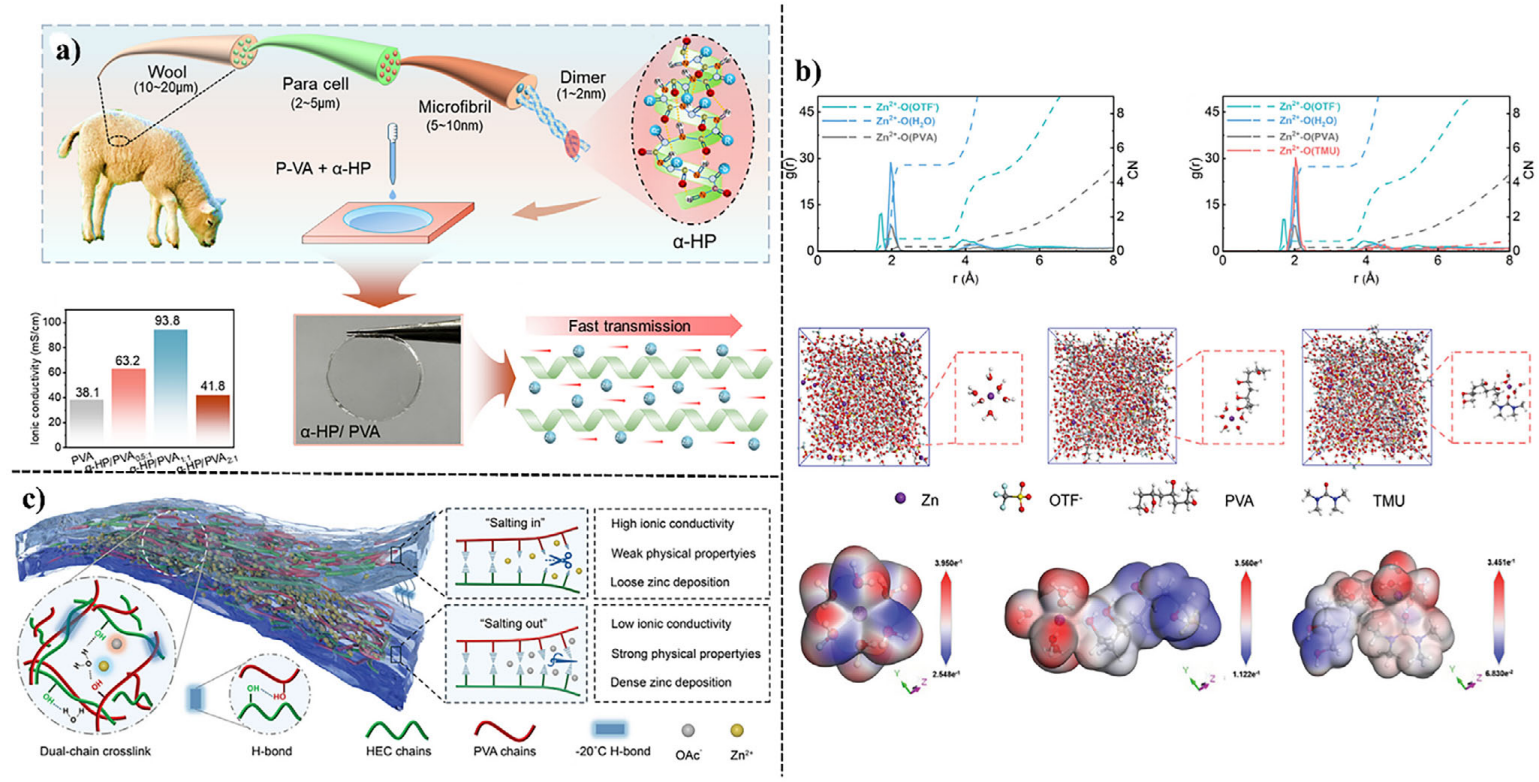
Representative design strategies for advanced PVA‐based hydrogel electrolytes. (a) α‐Helical protein/PVA hydrogel with interconnected pathways for rapid Zn^2+^ transport and enhanced ionic conductivity; reproduced with permission [32], 2024, RSC. (b) Molecular simulations and radial distribution functions illustrating TMU‐induced regulation of the Zn^2+^ solvation environment in PVA hydrogels; reproduced with permission [33], 2024, Wiley. (c) Concentration‐gradient PVA/HEC hydrogel constructed through salting‐in/salting‐out effects to balance ion transport, mechanical stability, and uniform Zn deposition; reproduced with permission [34], 2025, Wiley.

Molecular regulation of the hydrogen‐bond network and Zn^2+^ solvation structure provides another effective strategy for improving PVA‐based electrolytes. Tian et al. introduced tetramethylurea (TMU) into PVA, where its carbonyl groups participate in strong interactions with the polymer/water environment while the hydrophobic methyl‐containing moieties reduce free‐water activity. The molecular simulations and radial distribution functions shown in Figure 4b reveal pronounced interactions among Zn^2+^, water, PVA, OTf^−^, and TMU, demonstrating reconstruction of the Zn^2+^ coordination environment after TMU incorporation. The altered Zn^2+^ coordination environment reduced water‐associated interfacial reactions and facilitated more uniform Zn deposition, demonstrating the contribution of TMU‐mediated molecular modification to interfacial regulation. Thus, this study demonstrates that solvation‐structure engineering, rather than merely increasing hydrogel mechanical strength, can be used to actively regulate Zn interfacial chemistry [33]. Another innovative strategy for improving PVA‐based hydrogel electrolytes involves constructing spatially graded polymer networks rather than homogeneous hydrogel matrices. Zhou et al. developed a concentration‐gradient hydrogel electrolyte (CGHE) by integrating high‐ and low‐concentration zinc acetate layers within a dual‐network PVA/hydroxyethyl cellulose (HEC) hydrogel based on the Hofmeister effect. As schematically illustrated in Figure 4c, the Hofmeister‐effect‐induced “salting‐in” and “salting‐out” behaviors produce spatially differentiated polymer networks: the high‐salt region underwent a salting‐out process that strengthened hydrogen bonding and increased crosslinking density, providing excellent mechanical robustness, whereas the low‐salt region exhibited a salting‐in effect that preserved continuous hydrated ion pathways for rapid Zn^2+^ transport. The resulting gradient architecture effectively balanced ionic conductivity and mechanical strength, while simultaneously regulating Zn^2+^ flux and electric field distribution across the electrolyte. The spatially differentiated network balanced ionic transport and mechanical strength while improving Zn deposition uniformity and suppressing parasitic interfacial reactions. This work demonstrates that engineering spatial gradients in hydrogel electrolytes provides a new design paradigm for simultaneously optimizing ion transport, mechanical integrity, and interfacial stability in aqueous zinc‐ion batteries [34]. These characteristics represent the inherent advantages of PVA as a hydrogel matrix, whereas intelligent functionality arises from additional molecular or architectural modifications. In particular, biomolecular incorporation, solvation modifiers, and spatially heterogeneous network structures can actively regulate Zn^2+^ coordination, water activity, ion flux, and interfacial deposition. Thus, PVA provides the passive structural and transport framework, while the introduced functional components determine the extent to which the electrolyte can actively regulate electrochemical processes.

#### 3.1.2 Polyacrylamide Hydrogels

Polyacrylamide (PAM) is one of the most widely investigated polymer matrices for hydrogel electrolytes owing to its simple synthesis, excellent hydrophilicity, abundant amide groups, and highly tunable 3D network structure. The intrinsic advantages of PAM, including high electrolyte uptake, hydrophilicity, mechanical flexibility, and ionic transport, provide the passive foundation of the electrolyte. The amide‐rich PAM backbone supports interactions with electrolyte species, while its tunable network topology enables modifications to ionic transport, mechanical integrity, and interfacial stability. Accordingly, the following examples emphasize the influence of network architecture and functional additives on these properties. In addition to regulating Zn^2+^ solvation through functional groups, recent studies have shown that engineering the polymer network architecture is equally important for improving the performance of PAM hydrogel electrolytes [6, 35]. Shen et al. developed a highly entangled polyacrylamide (HE‐PAM) hydrogel by increasing the acrylamide monomer content while reducing the chemical crosslinker concentration, thereby transforming the conventional crosslink‐dominated PAM network into a structure dominated by long, physically entangled polymer chains. As illustrated in Figure 5a, the conventional C‐PAM network contains relatively disordered chains and localized crosslinking points that restrict continuous Zn^2+^ migration, whereas HE‐PAM forms highly interconnected and entangled chains that establish more continuous hydrated pathways for directional Zn^2+^ transport. The reduced chemical crosslink density decreases steric constraints on ion migration, while extensive chain entanglement preserves the structural integrity of the hydrogel. The interconnected, entangled chains distribute mechanical stress while maintaining continuous hydrated transport pathways. Consequently, HE‐PAM simultaneously achieves rapid Zn^2+^ transport, enhanced mechanical stability, and improved fast‐charging capability. This study demonstrates that polymer‐topology engineering can decouple the conventional trade‐off between ionic transport and mechanical robustness, providing an effective strategy for high‐performance PAM‐based hydrogel electrolytes [36].

**FIGURE 5:**
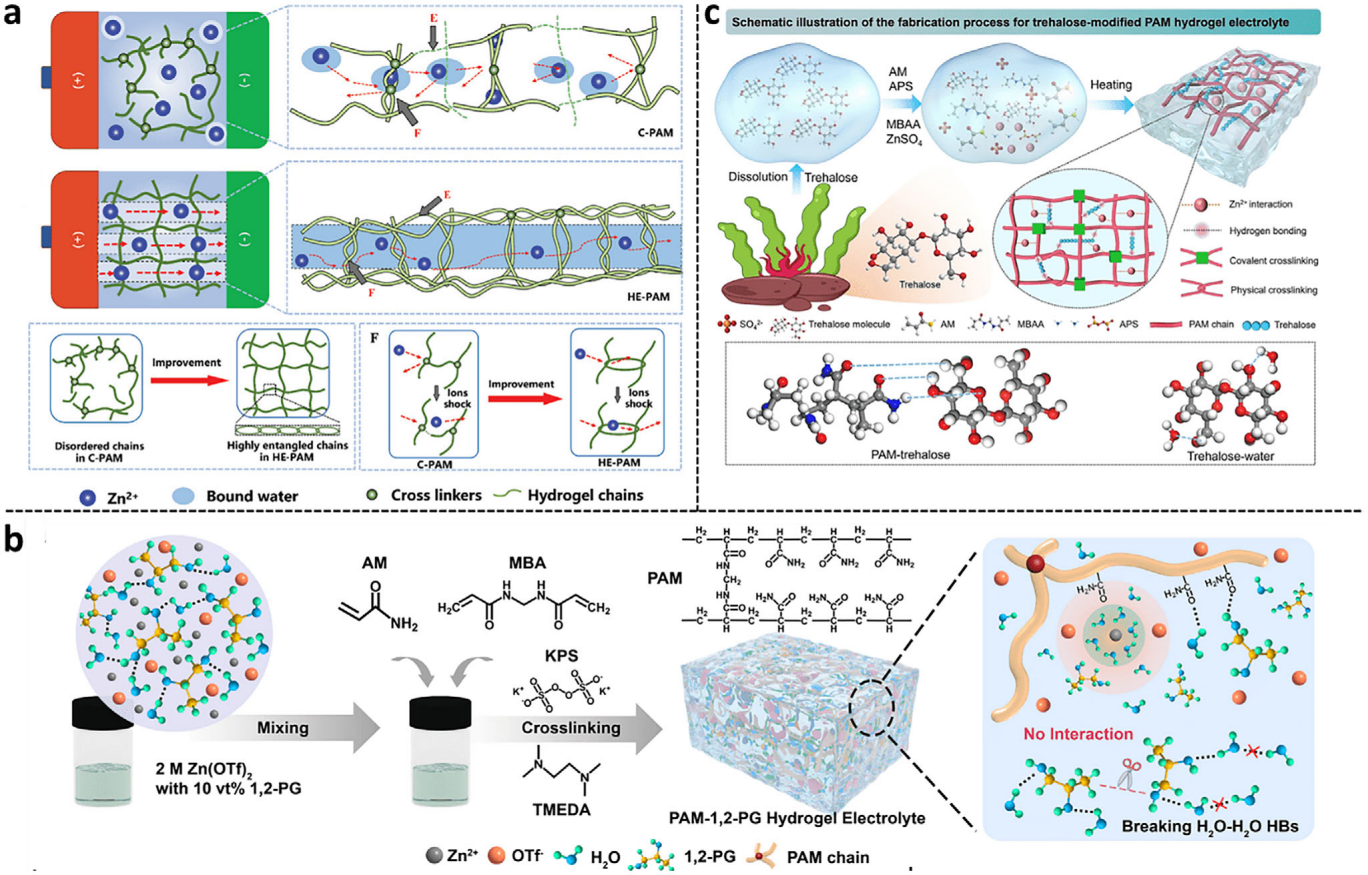
Representative network and molecular engineering strategies for PAM‐based hydrogel electrolytes. (a) Highly entangled PAM (HE‐PAM) network providing continuous Zn^2+^ transport pathways and enhanced resistance to ion‐induced perturbations compared with conventional crosslinked PAM; reproduced with permission [36], 2024, Wiley (b) 1,2‐PG‐modified PAM hydrogel showing disruption of the H_2_O–H_2_O hydrogen‐bond network and regulation of water activity; reproduced with permission [37], 2025, RSC (c) Trehalose‐modified PAM hydrogel illustrating hydrogen‐bond‐mediated physical crosslinking, network reinforcement, and interactions among PAM, trehalose, water, and Zn^2+^; reproduced with permission [38], 2024, Wiley.

Hydrogen‐bond network engineering has emerged as an effective strategy for enhancing PAM‐based hydrogel electrolytes. Guo et al. incorporated 1,2‐propylene glycol (1,2‐PG) into a PAM hydrogel containing Zn(OTf)_2_ to regulate the local water environment. As illustrated in Figure 5b, 1,2‐PG is uniformly incorporated into the crosslinked PAM network, where its hydroxyl groups interact strongly with surrounding water molecules, while the terminal methyl group interrupts the extended H_2_O–H_2_O hydrogen‐bond network through an “end‐capping” effect. This competitive hydrogen‐bonding interaction decreases the population and activity of free water without substantially increasing electrolyte viscosity, thereby maintaining efficient Zn^2+^ transport. In addition to modifying the local solvent environment, 1,2‐PG contributes to interfacial regulation and the formation of a protective hybrid interphase, thereby improving Zn deposition uniformity and reducing parasitic reactions. Moreover, disruption of the water–water hydrogen‐bond network inhibits ice crystallization, contributing to improved antifreezing capability and stable electrochemical performance over a wide temperature range [37].

Bio‐inspired molecular engineering provides an effective strategy for simultaneously strengthening PAM hydrogel networks and regulating their local ionic and water environments. Yang et al. introduced trehalose into a PAM hydrogel as a multifunctional network‐regulating agent. As illustrated in Figure 5c, trehalose is incorporated into the covalently crosslinked PAM framework and introduces additional physical crosslinking through extensive hydrogen‐bond interactions with the PAM chains. Trehalose introduces additional physical crosslinks within the covalent PAM framework, modifying network connectivity and improving structural robustness. Meanwhile, strong trehalose‐water interactions restructure the local hydrogen‐bond network and enhance water retention, while interactions between Zn^2+^ and functional groups within the PAM/trehalose network contribute to regulation of the ionic environment. The coexistence of covalent crosslinks, physical crosslinks, hydrogen bonding, and Zn^2+^ interactions therefore produce a reinforced yet adaptable hydrogel network. This molecular regulation improves electrode–electrolyte contact, homogenizes Zn^2+^ flux, and suppresses hydrogen evolution, Zn corrosion, by‐product formation, and dendritic growth. Furthermore, preferential Zn deposition along the stable (002) plane contributes to improved cycling stability and mechanical durability, demonstrating the effectiveness of bio‐inspired trehalose modification for constructing robust and flexible PAM‐based hydrogel electrolytes [38]. Engineering the microstructure of PAM hydrogels has emerged as an effective strategy to simultaneously improve ion transport, mechanical integrity, and interfacial stability. Liu et al. developed a homogeneously porous composite hydrogel electrolyte (CN‐PAM) by introducing alkali‐etched hydroxyl‐functionalized graphitic carbon nitride (OH^−^ g‐C_3_N_4_) nanosheets into a PAM network. The hydroxyl‐rich nanosheets function as multifunctional crosslinking centers that induce polymerization, strengthen the hydrogen‐bond network, and construct a highly uniform three‐dimensional porous framework. The interconnected porous channels shorten Zn^2+^ diffusion pathways, increase ionic transport density, and homogenize Zn^2+^ flux throughout the electrolyte, while the reinforced hydrogen‐bond network immobilizes free water molecules and suppresses parasitic reactions. Furthermore, the strong interaction between the hydroxyl‐functionalized nanosheets and Zn^2+^ partially reconstructs the Zn^2+^ solvation shell, reduces water coordination, and promotes uniform Zn deposition along the thermodynamically favorable (002) crystal plane. The synergistic regulation of pore architecture, hydrogen‐bond interactions, and Zn^2+^ solvation enables dendrite‐free Zn deposition, high ionic conductivity, enhanced mechanical robustness, and long‐term cycling stability, demonstrating that microstructure engineering represents an effective route for designing high‐performance PAM‐based hydrogel electrolytes [39]. PAM provides a versatile platform for architecture‐driven electrolyte design. Increasing chain entanglement reduces the dependence on dense chemical crosslinking, thereby maintaining transport pathways while improving mechanical integrity. In contrast, the incorporation of supramolecular crosslinkers and functional nanosheets introduces additional network junctions or interconnected porous structures. These approaches demonstrate that chain entanglement, crosslink density, and pore connectivity are key structural parameters governing the balance between ionic conductivity and mechanical robustness.

#### 3.1.3 Polyacrylic Acid Hydrogels

Poly(acrylic acid) (PAA)‐based hydrogels are typically prepared by free‐radical polymerization of acrylic acid, producing polymer chains rich in carboxylic acid (−COOH) groups. These groups constitute the principal ion‐regulating sites of the network because they can ionize to form carboxylate (−COO^−^) species and strongly interact with both water and Zn^2+^ [40]. Ionization increases local hydration and generates water‐rich domains that facilitate Zn^2+^ migration through the polymer matrix. Carboxylate groups provide reversible Zn^2+^ coordination sites, while polymer‐chain relaxation facilitates ion migration through the hydrated network. These characteristics enable PAA hydrogels to combine ion transport with interfacial regulation [41]. For PAA‐based hydrogels, water retention, ionic conductivity, and the mechanical integrity of the polymer network constitute the fundamental electrolyte characteristics, whereas the carboxylate‐rich chemistry provides an additional functional dimension. Zn^2+^ carboxylate coordination can reversibly alter the local solvation environment and regulate ion flux, while polymer to water interactions reduce the availability of free water. Further functionalization, such as inorganic reinforcement or metal‐ion coordination, can introduce enhanced interfacial stabilization, self‐healing, and environmental adaptability. Consequently, PAA‐based systems illustrate how an intrinsically ion‐conducting and water‐retaining polymer matrix can be converted into a multifunctional electrolyte through targeted chemical and network engineering.

A major limitation of pristine PAA hydrogels is their relatively low mechanical strength, which is often below 0.1 MPa. To address this weakness, Song et al. developed a PAA‐based composite gel polymer electrolyte (GPE) containing Al_2_O_3_ fillers for flexible ZABs. As shown in Figure 6a, pristine PAA exhibited a tensile strength of only 18.8 kPa, whereas incorporation of Al_2_O_3_ progressively strengthened the network through van der Waals interactions and hydrogen bonding with the polymer chains. Tensile strengths of 51.7, 104.5, and 190.2 kPa were reported for increasing Al_2_O_3_ contents. The 20 wt.% Al_2_O_3_ composition provided a balance between reinforcement and ionic transport, demonstrating the importance of optimizing filler loading rather than maximizing mechanical strength alone, delivering an ionic conductivity of 186 mS cm^−^^1^ and favorable electrolyte retention. The optimized PAA‐20% electrolyte also extended cycling to 384 h, compared with 127 h for pristine PAA (Figure 6b), and enabled a ZAB peak power density of 77.7 mW cm^−^^2^ [42]. These results illustrate the importance of optimizing filler content rather than maximizing mechanical reinforcement alone. Low‐temperature freezing and limited self‐healing capability represent additional barriers to the practical use of conventional gel polymer electrolytes. Lin et al. addressed these limitations by introducing Fe^3+^ ions into a cellulose‐nanofiber (CNF)‐reinforced PAA hydrogel. Reversible Fe^3+^–carboxylate coordination forms temporary supramolecular bridges between polymer chains, simultaneously increasing network strength and enabling self‐repair. The resulting Fe^3+^‐PAA/CNF hydrogel exhibited an approximately threefold increase in strength (up to 44.8 kPa) and an eightfold increase in toughness, while remaining flexible and unfrozen at temperatures down to −50°C. Its ionic conductivity decreased from 32 mS cm^−^^1^ at room temperature to 1.8 mS cm^−^^1^ at −40°C, yet remained sufficient to support low‐temperature electrochemical operation (Figure 6c,d) [43]. PAA can also regulate Zn deposition through its carboxyl‐rich chemistry. For example, Li et al. compared PAA, PAM, and polyethylene glycol additives in ZnSO_4_ electrolyte and found that PAA enabled stable Zn symmetric‐cell cycling for 360 h at 5 mA cm^−^^2^. The improved stability was attributed to carboxyl‐mediated suppression of dendrite formation, Zn corrosion, and parasitic side reactions [46]. PAA has further been applied to ZIHCs. Zhang et al. reported a quasi‐solid‐state zinc‐ion hybrid fiber capacitor using a ZnSO_4_‐filled PAA hydrogel, an rGO/CNT positive electrode, and Zn‐coated graphite fiber as the negative electrode (Figure 6e). The hydrogel exhibited an ionic conductivity of 21.7 mS cm^−^^1^, while the device delivered an energy density of 48.5 mWh cm^−^^3^ at a power density of 179.9 mW cm^−^^3^ [44]. Liu et al. developed a PAA‐based zwitterionic copolymer hydrogel through an ultrafast autocatalytic gelation process and subsequently incorporated sodium lignosulfonate (SL) and cellulose nanocrystals (CNCs) as reinforcing components. As shown in Figure 6f,g, the progressive introduction of SL and CNCs enhanced the mechanical properties of the hydrogel, with the optimized formulation reaching a toughness of approximately 800 kJ m^−^^3^. ZnCl_2_ was incorporated to reduce the hydrogel–electrode interfacial resistance, and the reinforcing additives did not substantially compromise the favorable ion‐transport characteristics of the electrolyte. The resulting ZIHC delivered 85.4 mAh g^−^^1^ at 1 A g^−^^1^ and retained 44.1 mAh g^−^^1^ at 10 A g^−^^1^; when the current density returned to 1 A g^−^^1^, the capacity recovered to 81.6 mAh g^−^^1^ (Figure 6h). Charge‐distribution analysis further confirmed the coexistence of diffusion‐controlled and capacitive storage contributions (Figure 6i) [45]. PAA‐based hydrogels demonstrate the importance of balancing chemical functionality with network reinforcement. Inorganic fillers improve mechanical strength through additional polymer‐filler interactions, whereas metal‐ion coordination introduces reversible junctions that can accommodate deformation. Incorporating cellulose nanocrystals provides an additional reinforcing framework that enhances toughness without substantially compromising ionic transport. Thus, filler dispersion, crosslinking mode, and the connectivity of reinforcing components are important design parameters for controlling the mechanical and transport properties of PAA hydrogels. The role of PAA‐based hydrogels depends strongly on the electrochemical system in which they are employed. In ZIBs, carboxylate groups can coordinate Zn^2+^ and modify its interfacial transport and deposition behavior. In ZABs, the effectiveness of PAA‐based networks additionally depends on their stability under strongly alkaline conditions and their influence on hydroxide and zincate transport. In ZIHCs, the electrolyte must retain sufficient ionic accessibility for both zinc‐electrode reactions and cathodic charge storage. Accordingly, the mechanical reinforcement and ion‐coordination effects observed in one device configuration should not be assumed to produce equivalent electrochemical benefits in another.

**FIGURE 6:**
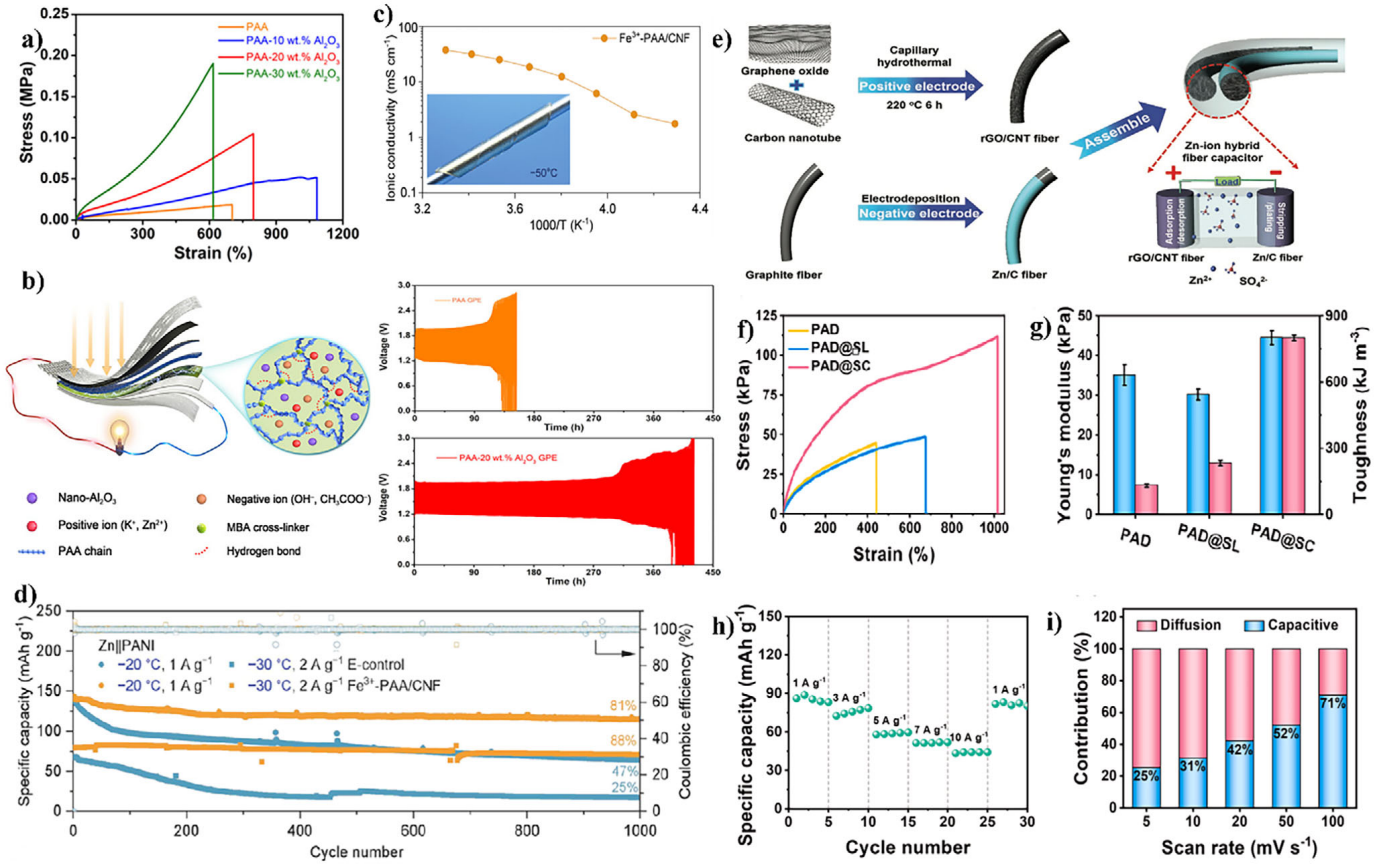
(a) Tensile stress–strain curves; (b) visual illustration of composite PAA‐Al_2_O_3_ and GCD profiles of PAA‐GPE and PAA‐20% Al_2_O_3_ GPE reproduced with permission.[42] Copyright 2022, ACS Publications. (c) Ionic conductivity of Fe^3+^‐PAA/CNF hydrogel at different temperatures, (d) long‐term stability analysis of Zn||PANI cells; reproduced with permission [43], 2025, Wiley. (e) Schematic illustration of the synthesis of rGO/CNT and Zn‐coated graphite fibers and the energy storage mechanism of ZIHFC; reproduced with permission [44], 2019, Wiley. (f) stress–strain curves, (g) Young's modulus and toughness results, (h) rate performance analysis, (i) charge distribution calculation reproduced with permission [45] 2024, Elsevier.

#### 3.1.4 Sodium Alginate Hydrogels

Sodium alginate (SA) is a naturally derived anionic polysaccharide composed of β‐D‐mannuronic acid (M‐block) and α‐L‐guluronic acid (G‐block) units distributed along the polymer backbone. Its abundant hydroxyl and carboxyl groups facilitate zinc‐salt uptake, ion dissociation, and coordination with Zn^2+^, while its low cost, biocompatibility, and film‐forming capability make SA an attractive matrix for hydrogel polymer electrolytes [47]. The intrinsic water uptake, ionic transport capability, and polymer‐network integrity of SA establish its basic electrolyte properties, whereas reversible metal–carboxylate coordination provides an adaptive functionality. The dynamic “egg‐box” network can simultaneously reinforce the polymer structure and permit reversible reconstruction after mechanical damage, illustrating how molecular interactions can convert mechanical support from a passive property into an adaptive self‐healing function. Additional modifications with cryoprotective solvents or chelating groups further extend this functionality toward antifreezing behavior, water‐activity regulation, and controlled Zn^2+^ solvation. Multivalent cation‐mediated crosslinking creates a three‐dimensional network with reversible junctions, providing mechanical reinforcement and the potential for self‐healing after damage [48]. This reversibility can impart self‐healing behavior after mechanical damage. Li et al., for example, reported a tough SA‐based hydrogel with a tensile strength of approximately 41 kPa and observable self‐healing after 12 h [49]. In electrochemical systems, Lu et al. developed a three‐dimensional double‐crosslinked gelatin/SA hydrogel electrolyte for flexible ZIBs. Compared with pure gelatin (GEM) and SA‐based hydrogels, the composite GAME electrolyte provided superior electrochemical stability. Zn symmetric cells using GEM failed after approximately 150 h at 0.5 mA cm^−^^2^, whereas GAME‐based cells remained stable for about 400 h without pronounced voltage fluctuations. In full cells using a V_2_O_5_/CNT cathode, GAME also outperformed the gelatin and liquid‐electrolyte controls, delivering 152 mAh g^−^^1^ at 20 A g^−^^1^ compared with 101 mAh g^−^^1^ for GEM. The device also maintained operation under bending, demonstrating the mechanical adaptability of the SA‐containing electrolyte [50].

Water activity remains a central limitation of SA‐based hydrogel electrolytes because free water promotes parasitic interfacial reactions, including hydrogen evolution at the Zn anode [51]. Lei et al. addressed this issue by modifying SA with a zinc citrate chelate derivative (ZCCD) through ion‐mediated crosslinking. Strong hydrogen‐bonding interactions reduced free‐water activity, while the chelating environment facilitated Zn^2+^ desolvation and decreased the probability of water‐driven side reactions. The combination of electrostatic interactions and metal‐coordination bonds also enhanced mechanical strength and ionic transport. The resulting Zn anode exhibited stable cycling for 5700 h at 0.5 mA cm^−^^2^ with a Coulombic efficiency of 99.6%, and the Zn||I2 full cell retained 79% of its initial capacity after 8000 cycles at 1.92 A g^−^^1^ [52]. Low‐temperature operation presents a second challenge because crystallization of the aqueous phase sharply decreases ionic conductivity and flexibility. Wang et al. incorporated ethylene glycol (EG) into a guar‐gum/SA matrix, obtaining an ionic conductivity of 6.19 mS cm^−^^1^ below −20°C and a specific capacity of 181.5 mAh g^−^^1^ at 0.1 A g^−^^1^ [47]. Gao et al. further developed antifreezing polycarboxylic double‐network electrolyte (SIP‐CS). Iminodiacetic acid was first grafted onto the SA backbone to increase carboxyl functionality, followed by copolymerization of acrylamide within the modified matrix. The resulting electrolyte was soaked in a ZnSO_4_/MnSO_4_/ZnCl_2_/sorbitol solution, where chloride and sorbitol acted as cryoprotective components. SIP‐CS exhibited an ionic conductivity of 14.36 mS cm^−^^1^ at −20°C, a fracture stress of 241.5 kPa, and a fracture elongation of 1011%; the corresponding Zn/SIP‐CS/MnO_2_ cell delivered 124.9 mAh g^−^^1^ at 1C at −20°C [53]. The network architecture of SA hydrogels is primarily governed by the arrangement of mannuronic‐ and guluronic‐acid units and the nature of their crosslinking interactions. Multivalent‐cation crosslinking produces mechanically reinforced networks, while incorporation of secondary polymers and cryoprotective components provides additional control over toughness, flexibility and temperature tolerance. The combination of natural polymer chains with double‐network or ion‐mediated crosslinking therefore offers a means of tailoring mechanical integrity and ionic transport for different device requirements.

### 3.2 Organohydrogel Electrolytes

Organohydrogels are polymeric networks that continue to provide the fundamental structural framework and ion‐conducting environment, while the incorporation of organic solvents introduces additional functional control over the water‐rich phase. Solvent–water and solvent–polymer interactions can reduce free‐water activity, suppress ice crystallization, retard evaporation, and reconstruct Zn^2+^ solvation structures. These effects extend the role of the electrolyte beyond passive water retention toward active environmental adaptation, particularly under subzero or elevated‐temperature conditions. Thus, organohydrogels represent an important transition from property‐oriented electrolyte design to multifunctional systems capable of regulating both electrochemical reactions and environmental stability. The water retention content in conventional hydrogels results in strong water activity, which promotes hydrogen evolution, zinc corrosion, and other parasitic reactions. Moreover, water crystallization at subzero temperatures severely reduces ionic conductivity and mechanical flexibility, whereas water evaporation at elevated temperatures accelerates electrolyte dehydration and performance degradation. These limitations restrict the operating temperature window and long‐term durability of hydrogel‐based zinc batteries [54]. To address these issues, organohydrogel electrolytes have emerged by partially replacing water with organic solvents such as dimethyl sulfoxide (DMSO), glycerol, ethylene glycol (EG), and other hydrogen‐bond‐active molecules. The incorporation of these solvents reconstructs the hydrogen‐bond network, regulates Zn^2+^ solvation, lowers water activity, and enhances water retention, thereby suppressing ice formation, electrolyte drying, and interfacial degradation while enabling stable ion transport over a wide temperature range [55, 56]. Recent advances have transformed organohydrogels from simple antifreezing media into multifunctional electrolyte platforms capable of simultaneously regulating ion transport, interfacial chemistry, and electrochemical stability through rational solvent engineering and polymer network design. Jiang et al. demonstrated this concept using a PAMPS/PAAm double‐network organohydrogel containing DMSO. Owing to its strong hydrogen‐bond‐accepting ability, DMSO preferentially interacted with water molecules, effectively reducing water activity, suppressing ice crystallization, and improving water retention. Consequently, the electrolyte maintained excellent flexibility and ionic conductivity at subzero temperatures while suppressing Zn corrosion, hydrogen evolution, and ZnO accumulation. In addition, DMSO altered the charging pathway in zinc‐air batteries by lowering the charging overpotential, illustrating that solvent engineering can simultaneously regulate both electrolyte stability and electrochemical reactions [57].

Another recent study by Qin et al. demonstrated that a polyanionic hydrogel electrolyte can actively regulate different water states at the Zn‐electrolyte interface. The engineered polyanionic environment simultaneously suppresses Zn^2+^‐solvated water deprotonation and free‐water ionization, thereby regulating hydrogen evolution and improving the reversibility of Zn chemistry. In addition, cooperative interactions within the polyanionic network facilitate Zn^2+^ desolvation and ion transport. This example demonstrates an experimentally validated form of intrinsic physicochemical regulation, in which the polymer electrolyte directly modulates interfacial water chemistry and parasitic reactions without requiring external sensing or control components [58]. Ethylene glycol has also been widely employed to improve the environmental adaptability of organohydrogels. Lyu et al. developed a PAM‐CMC‐EG organohydrogel in which EG reconstructed the hydrogen‐bond network while the zincophilic functional groups of PAM and CMC promoted uniform Zn deposition. The formation of mixed Zn(H_2_O)x(EG)γ^2+^ solvation structures reduced free‐water activity and homogenized Zn^2+^ transport, thereby suppressing hydrogen evolution, corrosion, and dendrite growth. Similarly, Wang et al. designed a PAMPS/PAAm organohydrogel using an EG/H_2_O binary solvent for flexible zinc‐ion hybrid supercapacitors. Strong interactions among EG, water, salts, and polymer chains significantly reduced ice crystallization and solvent evaporation, enabling stable operation from −30°C to 80°C. Meanwhile, the modified Zn interfacial environment promoted uniform Zn nucleation, inhibited by‐product formation, and delivered remarkable long‐term cycling stability under both low‐ and high‐temperature conditions. Beyond solvent regulation, bonding interaction engineering has emerged as an effective strategy for simultaneously controlling water activity, Zn^2+^ solvation, and interfacial deposition behavior [56]. Wei et al. developed a PAM/ZnSO_4_/glycerol/acetonitrile organohydrogel in which glycerol disrupted the water hydrogen‐bond network and partially replaced water molecules within the Zn^2+^ solvation sheath. Density functional theory calculations confirmed the formation of mixed Zn(H_2_O)m(GL)n^2+^ complexes that promoted uniform Zn deposition while reducing hydrogen evolution and sulfate‐based by‐products. Combined with the three‐dimensional ion conduction network and intimate electrolyte–electrode contact, the electrolyte effectively suppressed dendrite growth, enabling stable Zn||Zn cycling for over 3000 h and excellent Zn||V_2_O_5_ battery performance across a wide operating temperature range of −20°C–60°C. Collectively, these studies demonstrate that organohydrogel electrolytes extend far beyond conventional antifreezing agents. By simultaneously regulating hydrogen‐bond interactions, Zn^2+^ solvation structures, and electrode–electrolyte interfaces, they effectively suppress dendrite growth, reduce parasitic side reactions, and maintain high ionic conductivity under extreme environmental conditions. These multifunctional characteristics position organohydrogels as one of the most promising electrolyte platforms for next‐generation intelligent zinc‐based energy storage systems [59]. Although organic cosolvents can regulate water activity and suppress ice formation in all three device classes, their effects on charge transport are device‐dependent. In ZIBs, solvent‐induced changes in Zn^2+^ solvation and desolvation can influence deposition and cathodic ion insertion. In ZABs, the same solvent modifications must preserve adequate hydroxide mobility and zincate transport while maintaining the chemical stability required in alkaline environments. In ZIHCs, solvent engineering should also preserve rapid ion accessibility at the capacitive cathode. Consequently, improved anti‐freezing behavior or reduced water activity should not be considered sufficient evidence of an optimized electrolyte unless the relevant charge‐storage reactions and transport processes are also evaluated.

### 3.3 Composite Polymer Electrolytes

Composite polymer electrolytes (CPEs) are formed by incorporating a secondary phase such as inorganic nanoparticles, two‐dimensional nanosheets, clays, or carbon nanomaterials into a polymer matrix. This strategy is intended to overcome property limitations that are difficult to address with a single polymer component, particularly the trade‐off between ionic conductivity and mechanical robustness [60]. In aqueous zinc‐based systems, dispersed fillers can additionally serve as physical crosslinkers, water‐retention reservoirs, ion‐transport modulators, and interfacial regulators [61, 62]. According to filler chemistry, CPEs can be broadly divided into inorganic‐filler systems, including metal oxides and clays, and carbon‐based systems containing graphene derivatives, CNTs, or related conductive nanostructures. Composite polymer electrolytes provide a useful platform for separating passive reinforcement from active functionality. The polymer matrix primarily establishes the continuous electrolyte framework, solvent retention, and baseline mechanical integrity, while the secondary phase can introduce additional structural or functional roles depending on its chemistry. Inert fillers may predominantly reinforce the network or modify free volume and water retention, whereas ion‐active fillers can regulate Zn^2+^ transport, solvation, interfacial reactions, and local electric‐field distribution. Accordingly, the functional contribution of a filler should be distinguished from its simple effect on ionic conductivity or mechanical strength, because enhanced conductivity or reinforcement alone does not necessarily constitute intelligent electrolyte behavior.

#### 3.3.1 Inorganic Fillers

Inorganic fillers are among the most extensively studied modifiers for CPEs in zinc‐based energy‐storage systems. They include electrochemically inert oxide particles as well as ion‐active layered or two‐dimensional materials. Conventional oxides such as SiO_2_, Al_2_O_3_, TiO_2_, ZrO_2_, and ZnO can act as solid plasticizers: their high surface area and surface hydroxyl/oxygen groups disrupt polymer‐chain packing and crystallization, increase the amorphous fraction, and thereby facilitate ion transport [63]. TiO_2_ nanoparticles incorporated into PVA hydrogels, for example, have been reported to improve both room‐temperature ionic conductivity and mechanical strength [64]. Mesoporous SiO_2_ is particularly attractive because its nanoscale pore network can retain water while providing hydrated transport pathways. Hong et al. introduced SiO_2_ into PAM and achieved an ionic conductivity of 350.2 mS cm^−^^1^ [65]. ZnO has likewise been explored as a functional filler; Sun et al. combined DFT and experiment to show that optimized ZnO loading enhances low‐temperature Zn^2+^ transport and supports Zn symmetric‐cell cycling for more than 1500 h (Figure 7d) [66]. Chen et al. used a Keggin‐type polyoxometalate as both a crosslinking component and electrolyte modulator in a cellulose hydrogel. The resulting STA‐containing electrolyte reduced the Zn^2+^ hydration number and lowered the desolvation barrier, thereby improving interfacial transport. As summarized in Figure 7a–c, the liquid electrolyte exhibited a hydration number of approximately 5, whereas the STA hydrogel reduced this value to about 2 and delivered superior rate performance relative to pristine cellulose hydrogel and glass‐fiber controls [61]. Beyond these conventional oxides, MXenes and clays have also attracted researchers’ interest due to their high surface area and terminal functional groups, such as –O, –OH, and –F, allowing them to act as both mechanical reinforcement and ion‐conductive highways. For example, F. Huang et al. incorporated MXene into a PVA/Zn(CF_3_SO_3_)_2_ electrolyte to enhance ion‐transport pathways and promote the formation of a ZnF_2_ layer on the anode, suppressing dendrite formation. As a result, the flexible zinc‐ion batteries exhibited stable Zn stripping/plating and improved cycling stability for 2500 h at a current density of 1 mA cm^−^^2^ [67]. Beyond conventional oxide fillers, two‐dimensional nanomaterials and clays can provide simultaneous mechanical reinforcement and ion‐transport regulation. Tannic‐acid‐modified MXene nanosheets, for example, have been incorporated into PAM to establish three‐dimensional conductive pathways. The polymer network provides mechanical support, whereas the MXene phase contributes to ion‐conduction and interfacial regulation, producing an ionic conductivity of 27.69 mS cm^−^^1^ and a Zn^2+^ transference number of 0.833. The corresponding full‐cell maintained cycling for 10 000 cycles at 5 A g^−^^1^. Layered aluminosilicate and silicate clays, including Laponite and halloysite, are also attractive because of their high aspect ratio and negatively charged surfaces [68]. These clays can function as multifunctional physical crosslinkers and, when combined with conductive carbon phases, can improve ionic conductivity, mechanical strength, and self‐healing behavior [69].

**FIGURE 7:**
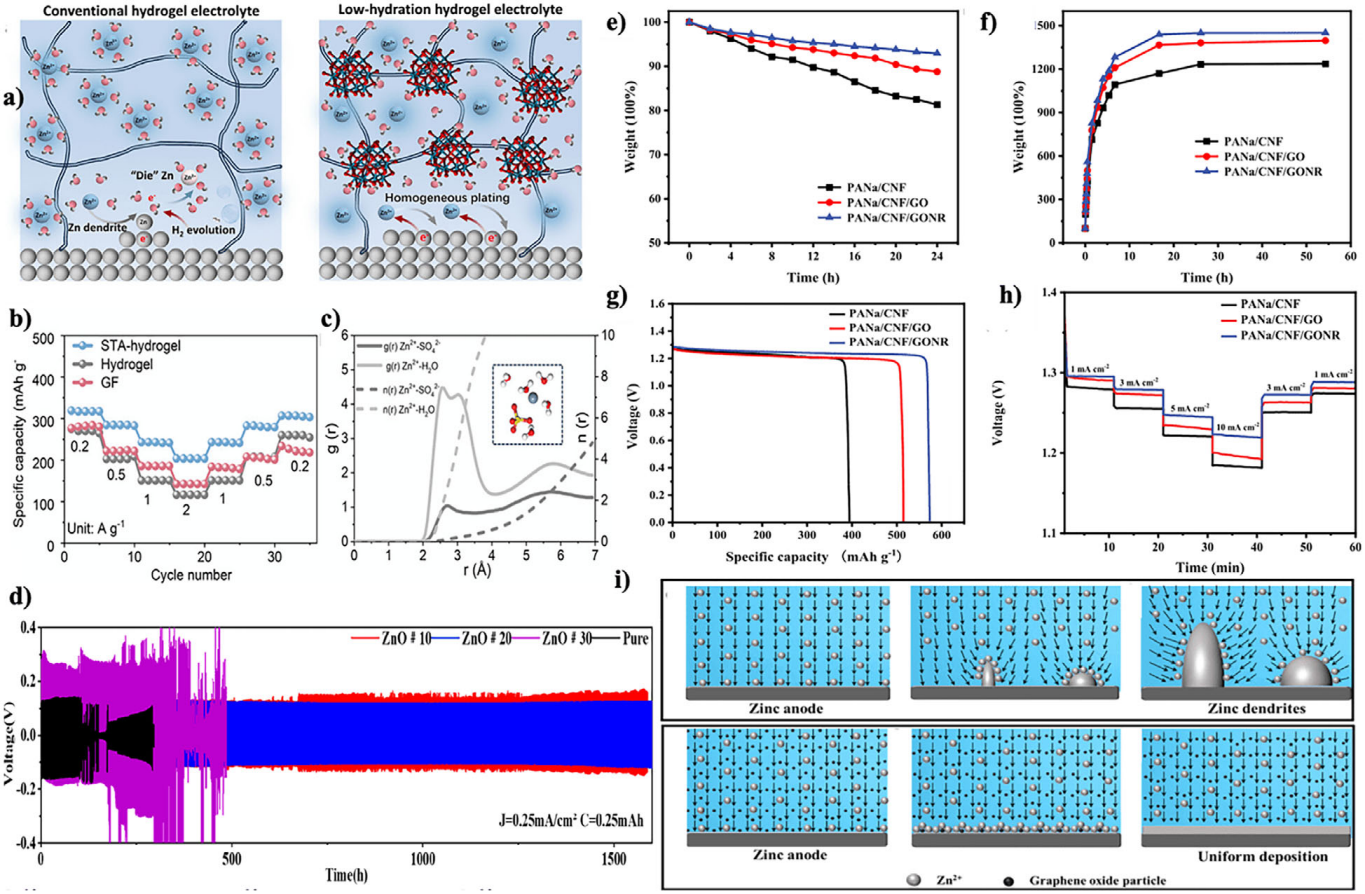
(a) Schematic illustration of Zn deposition using pure and composite polymers, (b) rate performance showing the highest specific capacity of CPEs, (c) DFT results for solvation of different electrolytes [61]. (d) cycling‐stability comparison of four electrolytes, reproduced with permission [66]. 2023, ACS Publications. (e) H_2_O retention capacity, (f) electrolyte uptake comparison, (g, h) discharge curves at constant (2 mA cm^−2^) and different current densities, respectively, reproduced with permission [70]. 2024, ACS Publications. (i) without and with GO electrolyte additive; reproduced with permission [71]. 2021, ACS Publications.

#### 3.3.2 Carbon‐Based Fillers

Carbon‐based fillers, including graphene oxide (GO), reduced graphene oxide (rGO), and carbon nanotubes (CNTs), differ from conventional inorganic fillers in that their electronic and interfacial properties can directly influence local electric‐field distributions at the electrolyte/Zn interface, in addition to providing mechanical reinforcement or water retention [72]. GO is particularly attractive because its oxygen‐containing functional groups provide abundant interaction sites for Zn^2+^. Suresh et al. combined computational and experimental analyses to show that reversible Zn–carbonyl association can regulate Zn^2+^ flux near the anode [73]. Incorporation of GO into a gel polymer electrolyte also homogenized the interfacial electric field and suppressed dendritic Zn growth (Figure 7i) [71]. Gu et al. similarly incorporated GO into an SA/PVA/PVP gel electrolyte and reported an ionic conductivity of 2.89 × 10^−^^3^ S cm^−^^1^ [74]. Liu et al. used graphene‐oxide nanoribbons (GONRs) together with cellulose nanofibers in sodium polyacrylate. The PANa/CNF/GONR composite achieved an ionic conductivity of 268.2 mS cm^−^^1^ and exhibited higher water retention and electrolyte uptake than the corresponding CNF‐ or GO‐containing controls (Figure 7e,f). This behavior was attributed to the high density of oxygen‐containing groups that stabilize water through hydrogen bonding. In a ZAB, the optimized PANa/CNF/GONR electrolyte delivered a specific capacity of 573.4 mAh g^−^^1^ and maintained higher discharge voltages across the tested current‐density range (Figure 7g,h) [70]. CNTs provide a complementary route to CPE reinforcement because their high aspect ratio can generate mechanically percolated and conductive networks at relatively low loading. This feature is particularly valuable in self‐healing and multifunctional hydrogel electrolytes [75]. Shang et al., for example, incorporated CNTs into a PAA‐AMPS hydrogel and obtained an ionic conductivity of 513.48 mS cm^−^^1^. The CNT‐containing network also suppressed parasitic reactions and promoted Zn^2+^ desolvation, enabling a flexible ZAB peak power density of 124.61 mW cm^−^^2^. In another system, CNTs incorporated into an acrylamide/PAA network produced a three‐dimensional composite hydrogel with an elongation at break of 506%, while the corresponding ZAB delivered a specific capacity of 677 mAh g^−^^1^. CNTs have also been combined with GO and Laponite, where carbon and clay fillers act cooperatively to improve ionic conductivity, tensile strength, and recoverability [76]. Overall, carbon‐based fillers provide interfacial electric‐field regulation and conductive network formation in addition to the structural reinforcement typically associated with inorganic fillers.

### 3.4 Solid‐State Polymer Electrolytes

Solid‐state polymer electrolytes (SPEs) differ fundamentally from hydrogels and organohydrogels because ion transport occurs predominantly through the polymer phase rather than through a water‐rich or solvent‐swollen network. By minimizing volatile or free liquid components, SPEs can eliminate electrolyte leakage, reduce solvent evaporation, and avoid freezing of an aqueous phase [77]. These characteristics are attractive for flexible and intrinsically safer Zn‐based devices, although solid polymer systems must still address Zn dendrite formation, interfacial instability, and limited room‐temperature conductivity [78]. Two principal transport concepts are commonly considered. In polymer hosts such as PEO, PAN, and poly(ethylene carbonate) (PEC), Zn^2+^ migration is coupled to segmental motion of the polymer chains, with cations hopping between coordinating oxygen or nitrile sites [79]. A second approach uses plastic‐crystal components such as succinonitrile (SN), whose orientationally disordered phase can support comparatively decoupled ion hopping through a quasi‐liquid sublattice [80]. More recently, Lu et al. developed a coordination polymer electrolyte based on a Lewis‐acidic deep eutectic environment in which polyacrylamide and acetamide ligands dynamically exchange around Zn^2+^. This ligand‐exchange process facilitated multivalent‐cation transport, yielding an ionic conductivity of 4.7 × 10^−^^3^ S cm^−^^1^ and a Zn^2+^ transference number of 0.44 [81]. A persistent limitation of SPEs is poor conformal contact with the Zn surface, which increases interfacial resistance. Zhao et al. addressed this issue using a Janus bilayer comprising PVDF‐HFP and PMMA. The dissimilar polymer layers generated a built‐in electric field and an interfacial ionic double layer that homogenized Zn^2+^ migration and deposition. Zn symmetric cells consequently operated for more than 13 000 h at room temperature, and the electrolyte remained functional from 28°C to 80°C [82]. Thus, although SPEs provide clear safety and environmental advantages, further improvements in low‐temperature segmental mobility and electrode–electrolyte contact are required to approach the ionic conductivity and interfacial compliance of hydrated polymer electrolytes.

Across the different polymer‐electrolyte platforms, ionic conductivity, water retention, and mechanical integrity constitute the fundamental properties required for an electrolyte to function as a stable ion‐conducting medium. Intelligent behavior emerges when these platforms are further engineered to perform active regulation or adaptive responses. Molecular coordination can regulate Zn^2+^ transport and solvation; polymer–water interactions can control water activity; interfacial interactions can stabilize Zn deposition and suppress parasitic reactions; dynamic bonds can enable self‐healing; and solvent or network engineering can provide antifreezing and temperature‐responsive behavior. The classification adopted here therefore distinguishes the structural identity of each electrolyte from the specific functional mechanisms responsible for its intelligent behavior. This distinction provides a consistent basis for evaluating the subsequent discussion of self‐healing, antifreezing, self‐adhesive, stretchable, and other adaptive electrolyte functionalities. Table 2 compares the major polymer‐electrolyte platforms discussed in Section 3 and shows various polymer electrolytes and their properties in different zinc‐based battery systems.

**TABLE 2:** Comparison of the major polymer‐electrolyte platforms and their properties in various zinc‐based battery applications.

ZABs					
Polymers Electrolyte	Synthesis Method	Ionic Conductivity	Tensile Strength	Electrolyte uptake/Retention	Ref
MF‐LDHs@PVA GPE	cross‐linking	138.87 mS cm^−1^ at 25°C	1200 kPa	94% after 24 h	[83]
Agar‐PVA/GO gel	freeze‐thaw cycling	75 mS cm^−1^ at 25°C	388 kPa	76.3% after 24 h	[84]
PAM‐PAA‐CNTs hydrogel	free radical polymerization	96.13 mS cm^−1^ at 25°C		490% after 24 h	[85]
COF@PAA gel	Free‐radical solution polymerization	290 mS cm^−1^ at 25°C	67 kPa	407% after 24 h	[86]
SA‐ PAAKS	free radical polymerization	59 mS cm^−1^ at 25°C	200 kPa	—‐	[87]
Organohydrogel Electrolyte	Free radical copolymerization	283.52 mS cm^−1^ at 25°C	6 kPa	615% after 24 h	[88]
**ZIBs**					
C‐CNFs—PVA	Hot‐melt casting ‐freeze‐thaw cycling	37.1 mS cm^−1^ at 25°C	207 kPa	58.19% after 24 h	[89]
PAM‐SL‐QCS	free‐radical copolymerization	28 mS cm^−1^ at 25°C	81 kPa	—‐	[90]
20%‐DPR/PAA	Free‐radical polymerization	23.24 mS cm^−1^ at 25°C	380 kPa	—‐	[91]
UiO‐66‐NH_2_ MOF‐	amidation grafting	38.1 mS cm^−1^ at 25°C	744.9 kPa	—‐	[92]
PAM/DMSO	free‐radical polymerization	77.8 mS cm^−1^ at 20°C	32.9 kPa	75.6% after 72 h (retention)	[93]
**ZIHCs**					
AA‐grafted PVA/CMC	free‐radical graft polymerization	42.2 mS cm^−1^ at 25°C	190 kPa	35.1% after 72 h (retention)	[94]
Carboxylated chitosan‐g‐PAM/PVA hydrogel	free‐radical polymerization	27 mS cm^−1^ at 25°C	69.6 kPa	—‐	[95]
PAM‐co‐PAA/*_K_*‐CG/ZnSO_4_	in situ UV photopolymerization	17.6 mS cm^−1^ at 25°C	42.5 kPa	390% after 12 h	[96]
SAP	In situ free‐radical polymerization	22.22 mS cm^−1^ at 25°C	70. kPa	—‐	[97]
PAMPS/PAAm	double‐network crosslinking	1 mS cm^−1^ at ‐30°C	0.22 MPa	—‐	[56]

## 4 Intelligent Functionalities

### 4.1 Self‐Healing Electrolytes

Mechanical damage remains a major challenge for flexible and wearable zinc‐based energy‐storage systems. Repeated bending, stretching, compression, or accidental cutting can produce cracks within the electrolyte, disrupt ion‐transport pathways, and cause interfacial separation between the electrolyte and electrodes. These changes increase internal resistance and may eventually lead to electrolyte leakage or device failure. Self‐healing electrolytes address these problems by incorporating reversible molecular interactions that can reconstruct the polymer network after damage and recover its mechanical integrity, ionic conductivity, and electrochemical functionality. The self‐healing process generally involves three consecutive stages: molecular contact between the damaged surfaces, diffusion or rearrangement of polymer chains across the interface, and reconstruction of reversible crosslinks. Depending on the nature of the reconstructing bonds, self‐healing electrolytes can be broadly classified into systems based on dynamic covalent bonds and those based on non‐covalent interactions. Dynamic covalent networks generally provide greater mechanical stability, whereas non‐covalent networks usually exhibit faster and more repeatable healing. Increasingly, these interactions are combined within hybrid or multilevel networks to balance mechanical strength, healing efficiency, ionic transport, and interfacial stability [16].

#### 4.1.1 Dynamic Covalent Bond‐Mediated Self‐Healing

Dynamic covalent bonds are reversible covalent linkages capable of dissociation, recombination, or bond exchange under appropriate conditions. Upon mechanical damage, dynamic bonds at the fractured interface are disrupted; subsequent contact between the damaged surfaces enables bond exchange and reconnection of polymer chains, thereby reconstructing the crosslinked network. Unlike permanent covalent networks, dynamic covalent systems therefore combine structural stability with repeated repair capability. Representative chemistries include boronate/boronic ester (B–O), imine (C = N), disulfide (S–S), acylhydrazone, oxime, and reversible Diels–Alder linkages. Their exchange kinetics can be controlled by temperature, water content, pH, light, or catalysts. Although these bonds generally provide greater structural stability than individual physical interactions, slow exchange kinetics or stimulus dependence can restrict healing under practical battery‐operating conditions. Among them, reversible B–O chemistry is particularly attractive for aqueous zinc electrolytes because dynamic exchange can occur effectively in water‐rich environments [98]. Puthiyaveetil et al. developed a PHBC‐4 hydrogel based on HPMC/PVA containing boric acid (BA), citric acid (CA), and Zn(CF_3_SO_3_)_2_, in which reversible B–O linkages provide the principal dynamic covalent framework. As illustrated in Figure 8a, mechanical cutting disrupts the dynamic crosslinks at the fractured interface, whereas subsequent contact enables re‐establishment of these reversible bonds and reconstruction of the hydrogel network. The healing process is further assisted by hydrogen bonding between the HPMC/PVA chains and reversible Zn–O coordination, generating a multilevel dynamic network that combines structural reinforcement with rapid molecular reorganization. The PHBC‐4 hydrogel consequently achieved approximately 93% healing efficiency within 5 min and also recovered its structural integrity following freezing–thawing treatment. Importantly, the recovered hydrogel could be integrated through in situ polymerization into a flexible Zn‐MnO battery configuration, demonstrating that dynamic network reconstruction can support both electrolyte recovery and practical device integration. Although multiple reversible interactions contribute to the healing process, the system can be classified primarily as a dynamic covalent electrolyte because reversible B–O bond reconstruction constitutes the key covalent healing mechanism [99].

**FIGURE 8:**
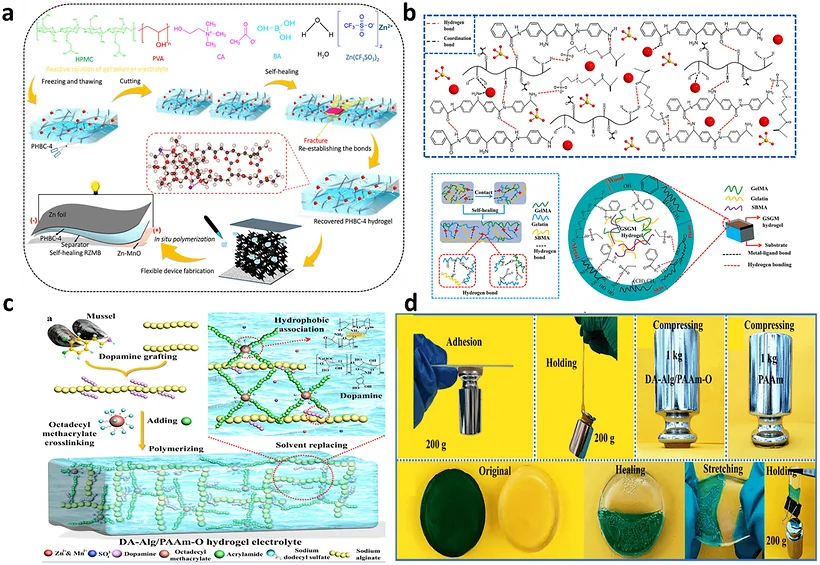
Representative dynamic covalent and non‐covalent self‐healing mechanisms in polymer electrolytes. (a) Dynamic B–O bond reconstruction in the PHBC‐4 hydrogel enabling rapid network recovery and flexible Zn–MnO battery integration; reproduced with permission [99], 2026, Wiley. (b) Cooperative hydrogen‐bonding, coordination, and electrostatic interactions enabling self‐healing and interfacial adhesion in the gelatin‐SBMA‐GelMA hydrogel; reproduced with permission [101], 2026, Elsevier. (c, d) Hydrophobic association and hydrogen‐bonding interactions in the DA‐Alg/PAAm‐O hydrogel, providing reversible network reconstruction, adhesion, and mechanical recovery; reproduced with permission [102] 2026, Elsevier.

Dynamic imine chemistry provides another versatile route for constructing self‐repairing electrolytes. Liao et al. reported a eutectogel in which reversible Schiff‐base reactions formed dynamic C = N bonds within the polymer network. When the electrolyte was damaged, imine‐bond exchange reconnected the fractured network, while hydrogen bonding and Zn^2+^ coordination promoted molecular contact and network rearrangement. The electrolyte achieved approximately 70% healing efficiency after 24 h at room temperature. This system illustrates the complementary functions of different reversible interactions: dynamic imine bonds restore network connectivity, while weaker physical interactions accelerate interfacial association and facilitate energy dissipation [100]. Despite these advantages, the presence of dynamic covalent bonds does not automatically guarantee effective electrolyte healing. Bond exchange reactions must proceed rapidly under the temperature, pH, and water content conditions experienced by the operating battery. Excessively high crosslinking density can restrict polymer chain diffusion and reduce healing efficiency, whereas insufficient crosslinking may lead to weak mechanical properties or electrolyte deformation. Dynamic covalent electrolyte design must therefore balance bond strength, exchange kinetics, segmental mobility, ionic conductivity, and electrochemical stability.

#### 4.1.2 Non‐Covalent Interaction‐Mediated Self‐Healing

In contrast to dynamic covalent systems, non‐covalent self‐healing relies on reversible supramolecular interactions that dissociate during damage and reassociate when the fractured surfaces are brought into contact. These interactions include hydrogen bonding, metal–ligand coordination, electrostatic and ion–dipole interactions, hydrophobic association, host–guest recognition, *π*–*π* stacking, and polymer‐chain entanglement. Although individually weaker than covalent bonds, their high reversibility and collective action facilitate rapid network reconstruction, often with lower activation barriers and without chemical bond‐exchange reactions. Figure 8b–d illustrates representative non‐covalent healing mechanisms based on cooperative hydrogen bonding/coordination and hydrophobic association, together with the resulting interfacial adhesion and mechanical recovery. Hydrogen bonding is the most widely employed non‐covalent mechanism because functional groups such as –OH, –NH_2_, –COOH, and C = O are abundant in conventional hydrogel polymers. Huang et al. developed an integrated Zn/PANI battery using a PVA/Zn(CF_3_SO_3_)_2_ hydrogel electrolyte. The hydroxyl groups of PVA formed a reversible intermolecular hydrogen‐bond network. Cutting disrupted these interactions, but recontacting the damaged surfaces enabled polymer chain diffusion and reformation of hydrogen bonds at room temperature without additional healing agents or external stimulation. The electrolyte exhibited an ionic conductivity of 12.6 mS cm^−^^1^ and maintained conductivity of the same order after ten cutting–healing cycles. The integrated battery retained approximately 81.4 mAh g^−^^1^ after three complete cutting–healing cycles, demonstrating simultaneous recovery of mechanical integrity and ion‐transport pathways [103]. A hydrogen‐bond‐mediated mechanism was also observed in the PAM/deep eutectic solvent DA‐ETG electrolyte. Multiple hydrogen bond donors and acceptors within the polyacrylamide and eutectic components created an extensive reversible physical network. After the fractured surfaces were contacted and heated at 40°C for 72 h, the hydrogen bonds reconstructed across the interface, allowing the healed electrolyte to support a 50 g weight. Although the required healing time was relatively long, this study illustrates how deep eutectic components can simultaneously participate in network reconstruction and provide a nonvolatile ion‐conducting environment [104].

Metal‐ligand coordination offers greater interaction strength while retaining dynamic reversibility. In the Fe^3+^‐PAA/CNF hydrogel, reversible coordination between Fe^3^+ ions and carboxylate groups on PAA and cellulose nanofibers acted as the main healing interaction. Hydrogen bonding provided additional support for network reconstruction. Following damage, coordination bonds near the fractured surface dissociated and subsequently reformed across the interface, producing approximately 95% self‐healing efficiency within 24 h at room temperature. Although metal‐ligand coordination may possess partial covalent character, it is generally categorized as a non‐covalent or supramolecular interaction in self‐healing polymer classifications because healing occurs through reversible ligand association rather than covalent bond exchange reactions [43]. Multiple non‐covalent interactions can operate cooperatively to provide both self‐healing and interfacial adaptability. In the gelatin‐SBMA‐GelMA electrolyte, abundant functional groups establish a dynamic supramolecular network through reversible hydrogen bonding, coordination, and electrostatic/ion‐dipole interactions. As depicted in Figure 8b, these reversible physical crosslinks can dissociate upon damage and re‐form after contact, while multipoint hydrogen‐bonding and metal–ligand interactions simultaneously promote adhesion to contacting substrates. Heating at 50°C enhances polymer‐chain mobility and facilitates rapid reconnection of the damaged interface within approximately 10 min. The same multipoint interactions also promote strong adhesion between the hydrogel and contacting substrates, contributing to stable electrode–electrolyte contact. Although the GelMA component provides a permanent covalent framework for mechanical reinforcement, it does not directly participate in the dynamic healing process. Therefore, the system is appropriately classified as a non‐covalent self‐healing electrolyte, with reversible supramolecular interactions primarily responsible for network reconstruction [101].

Hydrophobic association provides another effective non‐covalent crosslinking mechanism for constructing self‐healing polymer electrolytes. In the DA‐Alg/PAAm‐O hydrogel, dopamine‐grafted alginate and hydrophobic octadecyl methacrylate (OMA) segments form a dynamically crosslinked network through the cooperative action of reversible hydrophobic associations and hydrogen‐bonding interactions. As shown in Figure 8c,d, the hydrophobically associated network combines reversible reconstruction with strong adhesion and mechanical robustness, allowing the hydrogel to recover after cutting while retaining stretchability and load‐bearing capability. Cracks gradually disappeared within 24 h, with 72.4% mechanical healing efficiency retained after five healing cycles. More importantly, approximately 90.1% of ionic conductivity was recovered after 20 cycles, while the assembled battery retained 94.4% of its capacity following repeated cutting and healing. This distinction is important because effective electrolyte healing should restore not only macroscopic morphology but also continuous ion‐transport pathways and electrochemical functionality [102]. Not all mechanically recoverable electrolytes should be described as autonomous self‐healing systems. For example, the DMC‐PAM electrolyte recovered 98.52% of its maximum stress and 95.42% of its energy‐dissipation capability through hydrogen bonding, electrostatic interactions, and chain entanglement. However, these measurements primarily demonstrate recovery after deformation rather than autonomous reconnection of completely separated surfaces. Such systems are more accurately described as rapidly recoverable or fatigue‐resistant electrolytes unless cut‐contact‐heal experiments confirm complete network reconstruction.

#### 4.1.3 Hybrid Networks and Critical Considerations

The distinction between dynamic covalent and non‐covalent systems is not always absolute because advanced electrolytes frequently contain both interactions. In hybrid networks, dynamic covalent bonds provide permanent‐like structural reinforcement, while hydrogen bonding, coordination, or ionic interactions promote rapid surface contact, chain rearrangement, and energy dissipation. The PHBC‐4 and imine‐based eutectogel systems are representative examples of this strategy. Their classification should be determined by the interaction principally responsible for reconnecting the polymer network, while secondary interactions should be described as supporting mechanisms [99]. Electrolyte‐level healing should also be differentiated from self‐repair occurring exclusively within electrodes or artificial interfacial layers. For example, potential‐dependent adsorption and decomposition of PPS can continuously reconstruct a protective solid‐electrolyte interphase on Zn, but this process does not heal a damaged polymer electrolyte [105]. Similarly, polythioctic acid coatings can repair scratches through reversible hydrogen bonding while Zn–S bonds maintain coating adhesion; however, healing remains confined to the Zn protective layer. Supramolecular *π*–*π* stacking in perylene‐di‐imide electrodes likewise improves electrode organization and charge transfer without reconstructing the electrolyte. These studies may be briefly discussed as complementary interfacial self‐repair strategies but should not be presented as direct examples of self‐healing electrolytes [106]. Future studies should evaluate self‐healing performance using standardized and application‐relevant parameters. These should include healing temperature and duration, required external stimulation, mechanical healing efficiency, recovery of ionic conductivity, repeatability over multiple damage–healing cycles, interfacial adhesion, and electrochemical performance after healing. Visual disappearance of a crack alone does not demonstrate restoration of ion conduction or battery functionality. Full‐cell tests following repeated cutting, bending, or puncturing are therefore essential. The most promising self‐healing electrolytes will be those capable of rapid and autonomous repair at ambient or sub‐ambient temperatures while retaining high ionic conductivity, electrochemical stability, mechanical robustness, and compatibility with both Zn metal and the cathode [105].

### 4.2 Antifreezing Electrolytes

Subzero operation remains a major challenge for aqueous and hydrogel‐based zinc energy‐storage systems. Below 0°C, free water within the hydrogel can crystallize, disrupting hydrogen‐bonded channels that support Zn^2+^ transport. Ice formation sharply decreases ionic conductivity and induces volumetric expansion, which can crack the polymer matrix and destabilize the electrode–electrolyte interface [107]. Water confined within a hydrogel is commonly categorized as free, intermediate, or strongly bound water. Free water behaves most similarly to bulk water and is therefore most susceptible to crystallization; intermediate water is weakly associated with the polymer network; and strongly bound water interacts directly with polar groups along the polymer chains. Effective antifreezing design therefore aims to reduce the population and mobility of free water by reorganizing the hydrogen‐bond network. The principal strategies discussed below are solvent engineering, salt engineering, and polymer‐network engineering [108].

#### 4.2.1 Solvent Engineering

Solvent engineering introduces water‐miscible cosolvents such as ethylene glycol (EG), propylene glycol, or glycerol into the hydrogel phase to suppress water crystallization. Their hydroxyl groups compete for hydrogen bonding with water and disrupt the tetrahedral water network required for ice nucleation, thereby depressing the freezing point through combined colligative and specific intermolecular effects [37]. Chen et al. incorporated glycerol into a PVA hydrogel, where strong glycerol–water and glycerol–polymer interactions suppressed ice formation to approximately −60°C. The resulting flexible Zn–MnO_2_ battery exhibited an ionic conductivity of 10.1 mS cm^−^^1^ and stable operation at −35°C [109]. Wang et al. similarly introduced EG into a guar‐gum/SA matrix, obtaining an ionic conductivity of 6.19 mS cm^−^^1^ below −20°C and a specific capacity of 181.8 mAh g^−^^1^ at 0.1 A g^−^^1^ [47]. These examples demonstrate that cosolvent selection can simultaneously regulate the water hydrogen‐bond network, low‐temperature phase behavior, and ion transport. Beyond simple polyols, a combination of ethylene glycol and propylene glycol has been shown to reshape the internal H‐bond network of polyacrylamide (PAM) hydrogels. Their –OH and –CH_3_ groups disrupt extended H_2_O–H_2_O hydrogen‐bond chains, thereby improving low‐temperature stability and enabling stable Zn plating/stripping for more than 3780 h at −30°C [37]. Ou et al. fabricated a flexible battery by crosslinking a CO_2_‐derived cyclic carbonate within a potassium polyacrylate/polyacrylamide matrix. This system exhibited antifreezing behavior with intrinsic self‐healing properties, retaining 84% of the initial capacity at −20°C [110].

#### 4.2.2 Salt Engineering

Salt engineering suppresses freezing by strengthening ion–water interactions and disrupting the extended water–water hydrogen‐bond network. The magnitude of this effect depends on ion concentration, hydration strength, and ion‐specific interactions that are often interpreted in the context of the Hofmeister series. Yan et al. used concentrated ZnCl_2_/LiCl to reconstruct the aqueous hydrogen‐bond network, obtaining an ionic conductivity of 1.14 mS cm^−^^1^ and an activation energy of 0.21 eV at −50°C. A Zn//polyaniline cell retained nearly 100% of its initial capacity after 2000 cycles at this temperature [111]. Ion identity can further tune low‐temperature behavior: chaotropic anions such as ClO_4_^−^, CF_3_SO_3_^−^, and BF_4_^−^ perturb water organization, whereas highly charged cations such as Mg^2^+ strongly coordinate water oxygen atoms. Jiang et al. used a Zn(ClO_4_)_2_/Mg(ClO_4_)^2^ water‐in‐deep‐eutectic formulation to construct a hydrogel electrolyte with an ionic conductivity of 0.285 mS cm^−^1 at −70°C; the corresponding Zn–Mn cell delivered 76.83 mAh g^−^^1^ under subzero conditions [112]. Salt effects can also cooperate with polymer functional groups. Lin et al. showed that acetate anions and PAM amide groups jointly disrupt the water hydrogen‐bond network and suppress Zn corrosion and HER. Zn||Cu cells achieved an average Coulombic efficiency of 99.7% over 500 cycles from −20°C to 100°C, while a Zn//polyaniline cell retained 81.4% of its initial capacity after 1100 cycles at −20°C [113].

#### 4.2.3 Polymer Network Engineering

Polymer‐network engineering provides a third antifreezing strategy by directly controlling how water is confined within the crosslinked scaffold. Increasing the density of polar amide, hydroxyl, carboxyl, or sulfonic groups strengthens polymer–water interactions and shifts water from a free state toward intermediate or strongly bound states that are less susceptible to crystallization. These same functional groups can improve adhesion to the Zn surface and stabilize the electrode–electrolyte interface [108]. Huang et al. constructed a ternary hydrogen‐bonding network containing multiple donor and acceptor sites, thereby restricting long‐range ordering of water while maintaining ionic conduction at low temperature. Yan et al. combined cononsolvency and salting‐out effects to form a porous hydrogel with densely packed polymer chains and a strongly perturbed water network. The electrolyte remained functional at −77°C, exhibited a tensile strength of 15.6 MPa, and supported more than 30 000 cycles with reduced interfacial polarization [114]. UV‐cured polyacrylate networks containing hydroxyethyl acrylate (HEA), PVA, and Zn(CF_3_SO_3_)_2_ provide a related example in which abundant hydroxyl groups retain water and suppress crystallization while preserving flexible ZIB operation to −20°C. Collectively, these studies demonstrate that the polymer network itself can function as an active regulator of water phase behavior rather than merely as a mechanical scaffold.

### 4.3 Self‐Adhesive Electrolytes

Maintaining intimate electrode–electrolyte contact is essential for stable ion transport and interfacial electrochemistry in zinc‐based energy‐storage systems. However, repeated Zn plating/stripping, electrode volume changes, and mechanical deformation can induce interfacial gaps, slippage, or delamination, particularly in flexible devices. Such contact deterioration disrupts ion‐transport pathways, increases interfacial resistance, and may aggravate nonuniform Zn deposition. Self‐adhesive polymer electrolytes provide an effective solution by establishing persistent yet reversible interactions with electrode surfaces. Unlike conventional gels that primarily act as ion‐conducting media, adhesive electrolytes exploit functional groups such as –OH, –NH_2_, –COOH, –CONH_2_, and charged moieties to generate hydrogen bonding, electrostatic interactions, metal coordination, van der Waals forces, or physical interlocking at heterogeneous interfaces. An appropriate balance between bulk cohesion and interfacial adhesion is particularly important, as the electrolyte must remain mechanically robust while conforming closely to the electrode surface [115]. Hydrogen‐bond engineering represents one of the most straightforward approaches for achieving intrinsic adhesion. Li et al. developed a PAM‐tamarind seed polysaccharide–chitosan (PTC) hydrogel in which hydroxyl‐ and amino‐rich polysaccharides establish extensive multipoint hydrogen bonding with the PAM framework. Together with van der Waals forces and Zn^2+^ coordination, these interactions enabled reversible adhesion toward diverse substrates, including metals, glass, plastics, paper, and rubber. More importantly, the adhesive network suppressed interfacial slippage and preserved continuous ion transport pathways during deformation. The same hydrogen‐bond network also regulated the water state and reinforced the polymer matrix, resulting in an ionic conductivity of 51.3 mS cm^−^^1^ and a Zn^2+^ transference number of 0.887. Consequently, Zn//Zn cells operated for over 2900 h, while stable Zn plating/stripping was maintained for more than 580 h under bending conditions, demonstrating the importance of adhesive interfaces for mechanically robust zinc batteries [116].

Beyond hydrogen bonding, incorporating charged functional groups provides an effective route to strengthen adhesion while simultaneously regulating interfacial ion transport. Xu et al. developed a self‐adhesive polyzwitterionic PSAZn‐gel by copolymerizing sulfobetaine methacrylate with acrylamide. Cooperative hydrogen bonding, electrostatic interactions, and Zn‐related coordination endowed the electrolyte with strong adhesion toward different substrates, reaching approximately 108 kPa on Zn foil. Notably, the zwitterionic –SO_3_^−^ and quaternary ammonium groups were not limited to mechanical adhesion but also provided preferential pathways for ionic migration. Strong interactions between –SO_3_^−^ and Zn^2+^ facilitated Zn^2+^ desolvation and contributed to homogeneous deposition, while intimate electrolyte–electrode contact reduced contact and charge‐transfer resistance. The electrolyte consequently achieved an ionic conductivity of 59.0 mS cm^−^^1^, a Zn^2+^ transference number of 0.81, and stable Zn symmetric‐cell cycling for approximately 800 h. This study illustrates how adhesive functional groups can simultaneously act as interfacial anchors and ion‐regulating sites, integrating mechanical coupling with electrochemical interface stabilization [117]. The molecular balance between hydrophilic and hydrophobic interactions offers another strategy for tailoring adhesion. Li et al. constructed an amphiphilic hydrogel by combining hydrophilic 2‐hydroxyethyl acrylate with hydrophobic perfluorooctyl methacrylate. Optimizing the hydrophilic‐hydrophobic balance produced an adhesion strength of approximately 55 kPa and enabled spontaneous attachment to Zn foil, Cu foil, and carbon cloth. Interestingly, excessive hydrophobic content weakened adhesion by reducing swelling and limiting hydrogen bond formation with the substrate, highlighting that strong self‐adhesion requires an appropriate balance between interfacial bonding and internal network cohesion. The amphiphilic architecture additionally regulated the water hydrogen‐bond network and confined water within hydrophobic nanodomains, suppressing crystallization and enabling electrolyte operation at extremely low temperatures. Thus, the same molecular architecture simultaneously provided adhesion, interfacial regulation, and environmental adaptability, demonstrating the multifunctional potential of self‐adhesive electrolyte design [118].

More sophisticated adhesive electrolytes increasingly employ hierarchical interactions to combine strong adhesion with dynamic interfacial adaptability. Sun et al. developed a physically crosslinked PAA‐Fe^3+^‐CEC‐β‐CD (PFCD) hydrogel in which relatively strong Fe^3+^‐carboxylate coordination maintains network integrity, whereas reversible hydrogen bonds provide dynamic interfacial adaptability. Multiple interactions, including hydrogen bonding, coordination, electrostatic attraction, and physical interlocking, enabled strong adhesion toward Zn, carbon cloth, Ni, Al, and other substrates, with adhesion strengths of approximately 50 kPa on Zn and 48 kPa on carbon cloth. Importantly, this dynamic interface transformed discrete point contact into more conformal face‐to‐face contact, reducing interfacial voids and yielding an interfacial impedance of 14.1 Ω, close to that of the corresponding liquid‐electrolyte system. The adhesive interface remained intact even after 1000 bending cycles, demonstrating that dynamically re‐constructable interactions can continuously accommodate mechanical deformation while preserving electrochemical contact [119]. Collectively, these studies demonstrate that self‐adhesion should not be regarded merely as a mechanical characteristic of polymer electrolytes. Rationally designed adhesive interactions can maintain conformal electrode–electrolyte contact, minimize interfacial voids and delamination, and preserve continuous ion‐transport pathways during cycling and deformation. More importantly, the functional groups responsible for adhesion can simultaneously regulate Zn^2+^ transport, solvation/desolvation, water activity, and Zn deposition, transforming self‐adhesion from a passive mechanical property into an active interfacial‐regulation strategy. Future self‐adhesive electrolytes should therefore focus on balancing bulk cohesion with reversible surface interactions while ensuring that strong electrode binding does not impede ion mobility. Combining adhesion with complementary intelligent functions such as self‐healing, antifreezing behavior, and mechanical adaptability offers a promising route toward durable zinc‐based energy storage devices capable of maintaining stable electrochemical interfaces under practical operating conditions.

## 5 Intelligent Polymer Electrolytes for Zinc‐Ion Batteries

The performance of ZIBs is strongly influenced by the electrochemical environment established at the electrode–electrolyte interfaces. During repeated cycling, nonuniform Zn^2+^ transport and electric‐field distribution can induce dendritic Zn deposition, while active water promotes hydrogen evolution, corrosion, and interfacial by‐products. At the cathode side, dissolution of active species, local pH fluctuations, and insufficient electrolyte contact further contribute to capacity decay. These challenges become more pronounced under high Zn utilization, extreme temperatures, and mechanical deformation. Intelligent polymer electrolytes offer an effective approach to address these interconnected issues because their role extends beyond ion conduction. Through functional polymer networks, charged groups, catalytic centers, hydrophobic domains, and spatially regulated structures, these electrolytes can actively regulate Zn2+ transport, interfacial reactions, water activity, and electrode–electrolyte contact. Recent developments therefore demonstrate a transition from passive gel electrolytes toward functionally engineered systems capable of regulating both electrode interfaces and maintaining stable battery operation under demanding conditions.

### 5.1 Zn Anode and Interfacial Regulation

Stabilizing the Zn metal anode remains a primary objective in intelligent polymer electrolyte design. In conventional aqueous electrolytes, heterogeneous Zn^2+^ flux and electric‐field distribution promote localized deposition and amplify the tip effect, while direct Zn‐water contact accelerates HER and corrosion. Recent polymer electrolytes therefore aim to simultaneously regulate Zn^2+^ transport, nucleation, water activity, and interfacial electric fields rather than relying solely on mechanical suppression of dendrites. Hu et al. demonstrated this concept using an anhydrous PVDF‐HFP‐based gel polymer electrolyte containing an ionic liquid and immobilized Cu ions. Coordination between Cu ions and PAA provided catalytic sites that accelerated Zn^2+^ deposition and interfacial charge transfer, while the water‐deficient electrolyte environment reduced HER and corrosion. The optimized electrolyte exhibited an ionic conductivity of 24.32 mS cm^−^^1^, and the regulated Zn^2+^ kinetics promoted uniform deposition and suppressed dendritic growth [120]. This approach demonstrates that functional species incorporated into polymer electrolytes can actively modify Zn deposition kinetics rather than simply providing a physical barrier. Interfacial electric‐field regulation provides another effective route toward stable Zn deposition. Wang et al. developed a polycation‐regulated amphiphilic C18‐PCHA‐AAm hydrogel in which positively charged polymer chains regulate SO_4_^2^^−^ distribution and mitigate local electric‐field concentration. Meanwhile, hydrophobic C18 domains create a water‐deficient Zn interface, facilitating Zn^2+^ desolvation and reducing water‐induced reactions. These cooperative effects promote preferential Zn deposition along the stable (002) plane. The electrolyte achieved a Zn^2+^ transference number of 0.85 and an average Zn//Cu Coulombic efficiency of approximately 99.8% over 4928 h. More importantly, Zn//Zn cells maintained stable cycling at 51% Zn depth of discharge (DOD), indicating improved Zn utilization under more demanding conditions [121]. Moving beyond compositionally uniform electrolytes, Luo et al. introduced a gradient PVA@carbon‐dot hydrogel, in which negatively charged carbon dots migrate toward the Zn interface under an applied electric field. Operando electrochemical confocal microscopy directly visualized this redistribution, revealing dynamic enrichment of functional species near the electrode. The resulting gradient interface homogenized the electric field and Zn2+ flux, reduced the Zn nucleation barrier, and suppressed HER. Consequently, Zn//Zn cells operated for over 6500 h at 1 mA cm^−^^2^ and approximately 900 h at 85% Zn DOD [122]. Such spatially programmed electrolytes represent an important development toward adaptive interfaces in which bulk ion transport and interfacial Zn chemistry can be regulated independently. Together, these studies illustrate the evolution of Zn protection from passive dendrite blocking toward active control of Zn^2+^ transport, electric fields, water activity, nucleation, and crystallographic deposition.

### 5.2 Cathode–Electrolyte Compatibility and Full‐Cell Regulation

Although polymer electrolyte research has largely focused on the Zn anode, cathode‐electrolyte compatibility is equally important for achieving durable ZIBs. In quasi‐solid‐state systems, poor electrolyte penetration into porous cathodes can restrict active‐material utilization, while dissolution and local chemical changes during cycling further destabilize the cathode interface. Puthiyaveetil et al. addressed the contact limitation through in situ polymerization of a hydrogel electrolyte directly within a porous Zn‐doped MnO_2_ cathode. Unlike preformed gel membranes, the electrolyte precursor penetrated the three‐dimensional cathode structure before polymerization, generating intimate contact and continuous Zn^2+^ transport pathways throughout the electrode. This integrated interface improved active‐material utilization and reduced limitations associated with poor solid–solid contact. The resulting quasi‐solid‐state battery delivered an energy density of 214.14 Wh kg^−1^ and retained approximately 85% capacity after 1000 cycles, demonstrating the importance of controlling electrolyte formation directly at the cathode interface [123]. A broader strategy was demonstrated using a PAM/SC/CMC‐Na hydrogel capable of regulating both electrode interfaces. The polymer/citrate environment modifies Zn^2+^ coordination and reduces free‐water activity, producing homogeneous Zn transport with an ionic conductivity of 18.78 mS cm^−^^1^ and Zn^2+^ transference number of 0.83. At the cathode side, the electrolyte buffers local proton activity and coordinates dissolved Mn species, thereby suppressing Mn dissolution and stabilizing the MnO_2_ interface. The resulting Zn//MnO_2_ battery delivered 212.35 mAh g^−^^1^ at 0.2 A g^−^^1^ and retained 81.47% capacity after 1500 cycles. This dual‐interface approach highlights an important direction for intelligent electrolytes: stabilizing the Zn anode alone is insufficient when cathode degradation eventually becomes the dominant limitation [124].

### 5.3 Wide‐Temperature and Mechanically Adaptive ZIBs

Practical ZIBs may experience temperature fluctuations and mechanical deformation that conventional aqueous electrolytes cannot readily tolerate. Water crystallization at subzero temperatures restricts ion mobility, whereas elevated temperatures accelerate dehydration and parasitic reactions. Flexible batteries additionally require continuous electrolyte–electrode contact during bending, stretching, or compression. Temperature‐adaptive hydrogel electrolytes can simultaneously regulate water activity and Zn^2+^ transport to overcome the coupled limitations of parasitic reactions and sluggish ion kinetics at low temperatures. Ji et al. developed a component‐fluctuation‐modulated hydrogel electrolyte containing zwitterionic functional groups, which optimized the Zn^2+^ solvation structure by progressively replacing coordinated water with polymer functional groups. This regulation reduced water‐associated parasitic reactions while preserving Zn^2+^ transport through separated ionic migration channels. Importantly, the electrolyte maintained stable Zn plating/stripping for 3000 h at −20°C, demonstrating that intelligent polymer–ion interactions can provide temperature adaptability without sacrificing interfacial reversibility [125]. The PAM/SC/CMC‐Na hydrogel demonstrates how regulation of polymer‐water interactions can extend battery operation beyond ambient conditions. Reconstruction of the hydrogen‐bond environment increases the fraction of strongly confined water and improves water retention, while maintaining favorable Zn^2+^ transport. Zn symmetric cells consequently operated for approximately 937 h at −20°C, showing that molecular regulation of the electrolyte environment can simultaneously support interfacial stability and low‐temperature operation [124]. Liu et al. further demonstrated environmental and mechanical adaptability using a double‐network KC/SBMA‐co‐AAm hydrogel. The hydrogen‐bond‐rich network, maintained ion conduction across a broad temperature range, enabling the Zn‐MnO_2_ battery to deliver 228.67 mAh g^−^^1^ at −30°C, corresponding to approximately 90% of its room‐temperature capacity. Importantly, the flexible battery retained nearly 100% capacity during testing under 85% compression, while stable charge–discharge profiles were maintained at bending angles up to 135°. These results demonstrate that intelligent polymer electrolytes can integrate ionic conduction with mechanical compliance and environmental tolerance, which is particularly important for flexible and wearable ZIBs [126].

### 5.4 Toward Practically Relevant Intelligent ZIBs

Despite substantial progress, the practical value of intelligent polymer electrolytes cannot be judged solely from long Zn//Zn cycling under mild conditions. Symmetric‐cell lifetimes obtained at low areal capacities and shallow Zn utilization may not accurately represent the conditions encountered in practical full cells. Encouragingly, recent studies have begun evaluating electrolyte performance at substantially higher Zn utilization. The polycation‐regulated hydrogel enabled operation at 51% Zn DOD, while the gradient PVA@CD electrolyte sustained cycling at 85% DOD, indicating that intelligently regulated interfaces can remain effective even when a considerable fraction of the Zn reservoir participates in cycling [121, 122]. Future studies should therefore evaluate intelligent polymer electrolytes using more practically relevant parameters, including high cathode mass loading, limited Zn excess, thin electrolyte layers, reduced electrolyte content, high areal capacity, and long‐term full‐cell cycling. Equally important is demonstrating that intelligent functions remain effective under realistic environmental and mechanical conditions rather than only through isolated material tests. Overall, polymer electrolytes for ZIBs are evolving from passive ion‐conducting media toward active components capable of regulating the entire electrochemical environment. Catalytic centers can accelerate Zn^2+^ deposition, charged polymer chains can reconstruct interfacial electric fields, gradient structures can dynamically regulate Zn interfaces, in situ polymerization can improve cathode contact, and engineered polymer–water interactions can enable wide‐temperature and mechanically stable operation. Future progress will therefore depend on integrating these functions while maintaining high ionic conductivity, electrochemical stability, and practical manufacturability. Such system‐level regulation of ion transport, Zn deposition, cathode stability, water chemistry, and environmental adaptability will be essential for translating intelligent polymer electrolytes into durable and practically viable zinc‐ion batteries.

## 6 Intelligent Polymer Electrolytes for Zinc‐Air Batteries

Recent studies demonstrate a transition from hydrogels acting simply as electrolyte reservoirs toward intelligent multifunctional electrolytes that actively regulate the electrochemical environment. A representative strategy involves introducing functional ionic species into the polymer network to control zincate‐ion transport. Liang et al. incorporated chitosan quaternary ammonium salt (HACC) and La^3+^ as compound cationic additives into a PMA hydrogel. The positively charged HACC strongly interacts with Zn(OH)_4_^2^^−^, while competitive adsorption of La^3+^ at the Zn interface regulates electrodeposition and suppresses dendrites and ZnO by‐products. The optimized PMA‐HL_0.02_ electrolyte, operated in the alkaline Zn‐air battery electrolyte environment reported in the original study, achieved an ionic conductivity of 130.43 mS cm^−1^ and a reported zinc‐species transport number of 0.84. Here, the zinc‐species transport number refers to the transport contribution assigned by the original study to mobile zinc‐containing species, primarily zincate species under alkaline conditions, rather than to Zn^2+^ as a free cation. The corresponding flexible ZAB maintained high power densities of 113.2 and 93.65 mW cm^−2^ at −20°C and 80°C, respectively [127]. More sophisticated regulation can be achieved by constructing spatially differentiated hydrophilic/hydrophobic environments. Inspired by biological cell membranes, Zhang et al. designed an amphiphilic HEMA/DMAPS/MMA hydrogel (DHM), in which hydrophilic HEMA provides continuous transport channels and zwitterionic DMAPS exhibits strong affinity toward Zn species, whereas hydrophobic MMA establishes a water‐deficient region near the Zn surface. This combination reorganizes the Zn^2+^ solvation environment while restricting interfacial water activity, thereby simultaneously suppressing dendritic deposition and ZnO passivation. The DHM exhibited an ionic conductivity of 313 mS cm^−^^1^ and a reported zinc‐species transport number of 0.71, indicating preferential transport behavior of zinc‐containing species within the polymer electrolyte under the reported alkaline ZAB conditions. Importantly, this parameter should not be interpreted as a conventional Zn^2+^ transference number, because Zn^2+^ is not the dominant free zinc species in a strongly alkaline zinc‐air electrolyte; rather, soluble zincate species, predominantly Zn(OH)_4_^2−^, coexist with OH^−^ as the principal charge‐carrying species. The corresponding ZAB operated for 71 h at 2 mA cm^−2^ [128]. Water regulation is equally important for environmental adaptability. The PAZ‐HS hydrogel employs an ultraconcentrated 3 m Zn(Ac)_2_/14 m KAc electrolyte, corresponding to a total acetate concentration of approximately 20 m, in which acetate coordination around Zn^2+^ promotes conversion of free water into strongly bound water. Consequently, water activity is reduced, suppressing parasitic reactions while simultaneously limiting freezing and evaporation. The resulting electrolyte‐maintained Zn symmetric‐cell cycling beyond 160 h at −20°C, while the corresponding ZAB operated for more than 670 h at this temperature. Remarkably, series‐connected cells could power LEDs under environmental temperatures ranging from −80°C to 100°C, although reduced low‐temperature conductivity and increased polarization reveal the inherent trade‐off between strong water confinement and rapid ion transport [129].

Beyond solvation regulation, polymer architecture can create dedicated ion‐transport pathways. Incorporating sodium starch phosphate into PAM produced the PAS‐KI electrolyte, where phosphate groups regulate Zn^2+^ solvation while the ionic starch derivative homogenizes the electric field and ion distribution. Together with KI‐mediated cathodic redox kinetics, this electrolyte reached 252.5 mS cm^−^^1^ conductivity and enabled 752.2 mAh g^−^^1^ specific capacity, while maintaining operation from −50°C to 75°C. An even more explicit transport mechanism was demonstrated using a BC/PAM‐PMPTC double‐network hydrogel. The BC/PAM‐PMPTC electrolyte was evaluated under the alkaline ZAB electrolyte conditions reported in the original study, where KOH provides the alkaline medium and zinc acetate supplies the zinc‐containing species. In this system, OH^−^ transport is explicitly described through coupled vehicular and Grotthuss mechanisms, whereas Zn(OH)_4_^2−^ transport is associated primarily with zinc deposition and interfacial regulation. Their cooperative Vehicular‐Grotthuss mechanism produces an ultrahigh conductivity of 269.47 mS cm^−^^1^. Polarized functional groups additionally homogenize Zn(OH)_4_^2^^−^ flux and favor stable Zn(002) deposition, resulting in a peak power density of 89.76 mW cm^−^^2^ and operation from −45°C to 60°C [130]. Finally, intelligent electrolytes can integrate autonomous mechanical functions with electrochemical regulation. The physically crosslinked PAA‐Fe^3+^‐CEC‐β‐CD (PFCD) hydrogel combines Fe^3+^ coordination and reversible hydrogen bonding to provide mechanical resilience, adhesion, and self‐healing while maintaining porous pathways for ion and oxygen transport. Its uniform interfacial electric field suppresses the Zn tip effect and dendrite formation, enabling a flexible neutral ZAB with 59 mW cm^−^^2^ peak power density and stable operation for 500 h (3000 cycles) [119]. Collectively, these studies show that intelligent polymer electrolytes for ZABs are evolving from passive ion‐conducting matrices into adaptive interfacial regulators. Ionic functional groups, amphiphilic domains, water‐binding networks, directional transport pathways, dynamic interactions, and self‐healing architectures can collectively regulate Zn deposition, solvation, OH^−^ transport, water activity, mechanical integrity, and temperature tolerance. Future development should therefore prioritize integrating these functions without sacrificing conductivity, oxygen transport, or electrode kinetics, particularly under practically relevant current densities and prolonged environmental exposure.

Unlike mildly acidic ZIB electrolytes, alkaline ZAB electrolytes require simultaneous control of hydroxide and soluble zinc‐containing species. The transport of OH^−^ is essential for maintaining the kinetics of oxygen reduction and oxygen evolution at the air electrode, whereas zincate migration and its subsequent conversion to ZnO influence zinc utilization, electrode passivation, and the reversibility of zinc deposition. Therefore, an electrolyte exhibiting a high reported zinc‐species transport number does not necessarily demonstrate equivalent hydroxide transport or improved oxygen‐electrode kinetics. Moreover, restricting zincate migration excessively may adversely affect zinc redistribution and utilization, while excessive water activity can accelerate hydrogen evolution and corrosion. Polymer‐network engineering must consequently balance hydroxide conductivity, zincate mobility, water management, and alkaline chemical stability. The evaluation of these electrolytes should distinguish bulk ionic conductivity from zinc‐species transport selectivity and should include evidence of air‐electrode compatibility, zinc‐electrode reversibility, and resistance to carbonation during prolonged operation.

## 7 Intelligent Polymer Electrolytes for Zinc‐Ion Hybrid Capacitors

ZIHCs exhibit a hybrid electrochemical mechanism that imposes distinct requirements on polymer electrolytes. Unlike conventional ZIBs, in which cathodic charge storage is generally associated with Zn^2+^ insertion, extraction, or related redox processes, ZIHCs couple zinc plating/stripping at the negative electrode with capacitive ion adsorption and/or fast surface‐controlled pseudocapacitive reactions at the positive electrode. Consequently, electrolyte transport involves not only the delivery of Zn^2+^ to the zinc electrode but also the movement of ions responsible for charge compensation at the cathode. In ZnSO_4_‐based systems, sulfate‐containing species may contribute to the cathodic charge‐storage process, depending on the electrode material and mechanism. The relative contributions of cation transport, anion transport, and surface‐controlled reactions therefore depend on cathode chemistry and electrolyte composition. Polymer electrolytes designed for ZIHCs should consequently provide adequate ionic conductivity, maintain rapid ion accessibility at the porous cathode, and regulate Zn plating/stripping without imposing excessive transport resistance. An excessively restrictive polymer network may improve mechanical stability while limiting cathodic ion accessibility, illustrating the need to balance structural reinforcement with the transport requirements of both electrodes. ZIHCs typically combine a porous carbon, MXene, or conducting‐polymer cathode with a Zn metal anode [131, 132]. This asymmetric configuration couples battery‐type Zn plating/stripping with rapid capacitive or pseudocapacitive charge storage, enabling higher energy density than conventional supercapacitors while retaining high power capability and long cycle life. The hybrid mechanism, however, imposes demanding electrolyte requirements. At the anode, the electrolyte must support rapid and spatially uniform Zn^2+^ desolvation, transport, and deposition; at the cathode, it must maintain fast and reversible access of the relevant ionic species to high‐surface‐area adsorption and redox sites. A sufficiently wide electrochemical stability window is also required to maximize device voltage [133]. Conventional liquid electrolytes are additionally susceptible to leakage, parasitic reactions, dendrite formation, and freezing, motivating the development of intelligent polymer electrolytes specifically tailored to ZIHC operation. Antifreezing and self‐adhesive hydrogel electrolytes are particularly relevant to flexible ZIHCs intended for wearable or outdoor electronics. Fu et al. developed a self‐adhesive polyzwitterionic hydrogel in which zwitterionic side chains simultaneously inhibited ice crystallization and maintained reversible adhesion to the electrodes. The resulting ZIHC preserved electrochemical and mechanical performance under subzero conditions without interfacial delamination [134]. Nan et al. combined a chemically crosslinked polymer network with concentrated ZnCl_2_/LiCl salts to obtain a mechanically robust low‐temperature electrolyte. A Zn//CNT hybrid capacitor delivered an energy density of 39 Wh kg^−^^1^ at −60°C, retained 98.7% of its initial capacity after 10 000 cycles, and remained flexible during repeated bending at that temperature [135]. Mechanical damage represents a second challenge because the hydrogel often functions simultaneously as electrolyte, separator, and structural component in flexible devices. Self‐healing networks can restore mechanical continuity and ion‐transport pathways after cutting, tearing, or repeated deformation [136]. Liu et al. developed a PVA/CMC‐Na hydrogel electrolyte with self‐healing capability and an ionic conductivity of 2.37 mS cm^−^^1^ at −20°C. The corresponding ZIHC delivered areal capacitances of 270.4 mF cm^−^^2^ at room temperature and 61.7 mF cm^−^^2^ at −20°C, while retaining 81.7% capacity after 10 000 cycles [137]. Gao et al. further combined Schiff‐base chemistry, hydrogen bonding, and metal coordination in a PVA/poly(N‐(2‐hydroxyethyl)acrylamide) double network. The electrolyte exhibited an ionic conductivity of 3.7 mS cm^−^^1^ at −30°C, a tensile strength of 1.85 MPa, and 760% elongation. After fracture, 90 s of near‐infrared irradiation restored approximately 83% of the original tensile strain and nearly 100% of the ionic conductivity [138].

Solvation and interfacial regulation provide a complementary strategy for extending ZIHC lifetime. This is particularly important because the porous cathode can often sustain very large cycle numbers, whereas instability of the Zn metal anode becomes the dominant failure mode. Dong et al. developed a LiCl‐containing dual‐network PAM/PVA hydrogel (PPZL) in which amide and hydroxyl groups reorganize the Zn^2+^ solvation sheath, while Li^+^ promotes the formation of a Li_2_O‐rich interfacial layer. This coupled solvation/interphase regulation enabled Zn||Zn symmetric cells to cycle for approximately 8000 h and an assembled ZIHC to operate for more than 100 000 cycles [139]. Xie et al. used a polyanionic hydrogel formed by copolymerizing 2‐acrylamido‐2‐methylpropane sulfonic acid (AMPS) with hydroxymethyl acrylate (HEA). Fixed sulfonate‐rich domains create preferential Zn^2+^ migration pathways and perturb the hydrated Zn^2+^ solvation shell, thereby promoting more uniform cation transport and stabilizing the Zn interface [140]. These studies highlight that long‐lived ZIHCs require electrolyte designs that address both rapid cathodic ion access and the much more stringent reversibility demands of the Zn anode. A device‐level comparison of various polymer electrolytes in various zinc‐based battery system is shown in Table 3.

**TABLE 3:** Device‐specific requirements and representative intelligent‐polymer strategies for major zinc‐based energy‐storage systems.

ZIHCs					
Polymer electrolyte	Cathode	Capacity	Capacity Retention	Stability	Ref
Poly(SBMA‐co‐AA)	Activated carbon	253 mAh g^−1^ at 0.5 A g^−1^	69.5% at 20 A g^−1^	95.6% (100 000 cycles at 5 A g^−1^)	[134]
(PHCD‐Zn)	Activated carbon	67.3 mAh^−1^ g at 5 A g^−1^	44.6% at 10 A g^−1^	85.4% (21 700 cycles at 5 A g^−1^)	[141]
AF PVA‐CMC/Zn(CF_3_SO_3_)_2_	Polymer‐derived carbon	107.9 mAh g^−1^ at 0.25 A g^−1^	27.7% at 15.0 A g^−1^	84.8% (10 000 cycles at 2.0 A g^−1^)	[142]
PAMPS/PAAm	Activated carbon	129.1 Fg^−1^ at 0.2 Ag^−1^	**42.1%** at 10 Ag^−1^	90.2% (100 000 cycles at 2 A g^−1^)	[56]
CBH‐Zn	Activated carbon	301.6 F g^−1^ at 0.5 A g^−1^	54.6% at 20.0 A g^−1^	94% (45 000 cycles at 10.0 A g^−1^)	[21]

ZABs					
Polymer electrolyte	Cathode	Discharge capacity/Duration	Power Density	Stability	Ref
nano‐SFQ	Co_3_O_4_	242.23 mAh cm^−3^ at 1 mA cm^−2^	104.2 mW cm^−2^	450 h at 1 mA cm^−2^	[143]
PVA/N‐PAM/KOH	MnO_2_/Co_3_O_4_	675 min at 2.0 mA cm^−2^	40.43 mW cm^−2^	113 cycles at 2.0 mA cm^−2^	[144]
AAS25‐SA‐GPE	Air cathode	760 mAh g^−1^ at 1 mA cm^−2^	170.9 mW cm^−2^	1200 cycles at 0.5 mA cm^−2^	[42]
PAA‐20 wt.% Al_2_O_3_ GPE	Co_3_O_4_‐Ni‐Mesh	2280 min at 2.0 mA cm^−2^	77.7 mW cm^−2^	384 h at 2 mA cm^−2^	[60]
5 wt.% silica/PAM	Co_3_O_4_	4500 min at 3.0 mA cm^−2^	162.8 mW cm^−2^	**115 h at** 3 mA cm^−2^	[145]

ZIBs					
Polymer electrolyte	Cathode	Specific capacity	Capacity Retention	Stability	Ref
(TA‐PVA)	α‐MnO_2_	151 mAh g^−1^ at 0.5C	54.8 mAh g^−1^ at 5.0C	151 mAh g^−1^ (100 cycles at 0.5C)	[146]
PuL‐PV‐PPZ	PANI/CC	211.25 mAh g^−1^ at 0.1 mA cm^−2^	**2 mA cm^−2^** at **47.3%**	87.42% (6000 cycles at 2 mA cm^−2^)	[147]
o‐PAM	VO_2_	195 mAh g^−1^ at 0.25 A g^−1^	**58.5%** at 10 A g^−1^	85.8% (1500 cycles at 5 A g^−1^)	[148]
PAN/PAM/SA	V_2_O_5_	409 mAh g^−1^ at 0.1 A g^−1^	66.3% at 5 A g^−1^	86.1% (1000 cycles at 5 A g^−1^)	[149]
PVEMA	V_6_O_13_	280.2 mAh g^−1^ at 0.5 A g^−1^	48.4% at 7 A g^−1^	92.04% (4000 cycles at 5 A g^−1^)	[150]
CMC‐Borax	MnO_2_	177.57 mAh g^−1^ at 0.2 A g^−1^	47.2% at 5 A g^−1^	90.05% (500 cycles at 1.8 A g^−1^)	[151]
SA‐CMC	Na‐V_2_O_5_	360 mAh g^−1^ at 3 A g^−1^	77% at 5 A g^−1^	68.2% (1000 cycles at 3 A g^−1^)	[152]

## 8 Computational and AI/ML‐Assisted Design of Intelligent Polymer Electrolytes

The molecular and structural complexity of polymer electrolytes makes purely experimental optimization inefficient because ionic conductivity, Zn^2+^ transport, solvation, water activity, mechanical integrity, and electrode‐electrolyte interactions are strongly coupled. Computational approaches provide complementary routes for understanding and accelerating electrolyte development across different length and time scales. Quantum‐mechanical methods, particularly density functional theory (DFT), can elucidate molecular interactions, ion coordination, adsorption energies, reaction pathways, and interfacial energetics at the atomic scale, while ab initio molecular dynamics (AIMD) can capture the dynamic evolution of ion–solvent and ion–polymer interactions. Classical molecular dynamics (cMD) extends these investigations to larger polymer/electrolyte systems and longer timescales, enabling analysis of ion diffusion, coordination environments, radial distribution functions, and transport pathways. In parallel, artificial intelligence and machine learning (AI/ML) methods can extract structure–property relationships from experimental or computational datasets and accelerate candidate screening and formulation optimization. These approaches are complementary rather than interchangeable: DFT and AIMD provide molecular‐level mechanistic information, cMD enables mesoscale transport analysis, and AI/ML facilitates rapid prediction across multidimensional composition–property spaces. For intelligent polymer electrolytes, particularly those designed for aqueous zinc‐based batteries, combining these approaches with experimental validation provides a pathway toward mechanism‐guided and data‐driven electrolyte design. A conventional workflow typically involves iterative synthesis of candidate electrolytes, physicochemical characterization by spectroscopic and microscopic techniques, and electrochemical evaluation using symmetric or full cells to determine parameters such as impedance, ionic conductivity, and Coulombic efficiency. This sequential trial‐and‐error process can substantially limit the number of formulations explored. Machine learning (ML) and artificial intelligence (AI) offer a complementary strategy by extracting structure–property relationships from experimental and computational data and using them to prioritize promising candidates before synthesis [153]. Properly trained ML models can therefore reduce the experimental search space, identify multi‐parameter trade‐offs, and guide formulation toward predefined performance targets. Although data scarcity, descriptor selection, and model transferability remain important challenges, AI/ML methods have already been applied successfully to polymer solar cells, separation membranes, conductive polymers, and polymer dielectrics [154].

### 8.1 Machine Learning for Polymer Electrolyte Designs

AI/ML methods have recently emerged as complementary tools for accelerating polymer‐electrolyte discovery by establishing quantitative relationships between polymer chemistry, electrolyte composition, processing conditions, and electrochemical properties. Although several pioneering computational studies have focused on Li^+^‐conducting polymer electrolytes, their methodological principles are relevant to zinc‐based systems but should not be considered directly equivalent because Zn^2+^ transport involves stronger ion–polymer interactions, multivalent‐ion coordination, aqueous solvation, and coupled interfacial reactions. Therefore, greater emphasis should be placed on ML studies specifically addressing Zn^2+^‐containing polymer electrolyte systems. Compared with inorganic solid electrolytes, ML applications to polymer electrolytes remain relatively underdeveloped, largely because polymer systems must simultaneously satisfy coupled requirements for ionic conductivity, mechanical modulus, processability, and electrochemical stability, while consistent polymer‐property datasets remain limited. Representative studies include the transfer‐learned graph neural network of Hatakeyama‐Sato et al. for Li^+^‐conducting polymer electrolytes [156] and the multi‐property screening framework of Xie et al. across thousands of amorphous polymers [157]. For aqueous Zn systems, Zhu et al. demonstrated that useful ML guidance can be obtained even from a small, exclusively experimental dataset [155]. Their workflow (Figure 9a) used support vector regression and random forest models trained on a dataset of 1200 experimental data points randomly split into an 80:20 training‐to‐test ratio consisting of 960 training points and 240 test points, with feature scaling performed using Standard Scaler across 11 retained experimental features, including electrochemical variables and electrolyte/polymer concentrations, to predict ionic conductivity and corrosion‐related behavior of PAM‐based GPEs with different entanglement densities. Feature‐importance analysis (Figure 9b1,b2) identified the variables most strongly associated with AC impedance and corrosion rate, while parity plots showed close agreement between predicted and measured values (Figure 9c,d). The resulting composition property map (Figure 9e) revealed a useful design trade‐off: increasing PAM concentration caused only a gradual decrease in ionic conductivity but a substantially stronger reduction in corrosion current. This analysis identified a highly entangled PAM network as an advantageous regime in which interfacial water activity and corrosion could be suppressed without severely compromising ion transport. Guided by the ML predictions, the optimized PAM‐based GPE with Zn(ClO_4_)_2_ achieved a Zn||Cu Coulombic efficiency of 99.63% over 1600 plating/stripping cycles and enabled more than 9000 cycles in a Zn||MnO_2_ full cell. This study demonstrates that ML can meaningfully reduce the experimental burden of multi‐parameter GPE optimization even when large simulated databases are unavailable.

**FIGURE 9:**
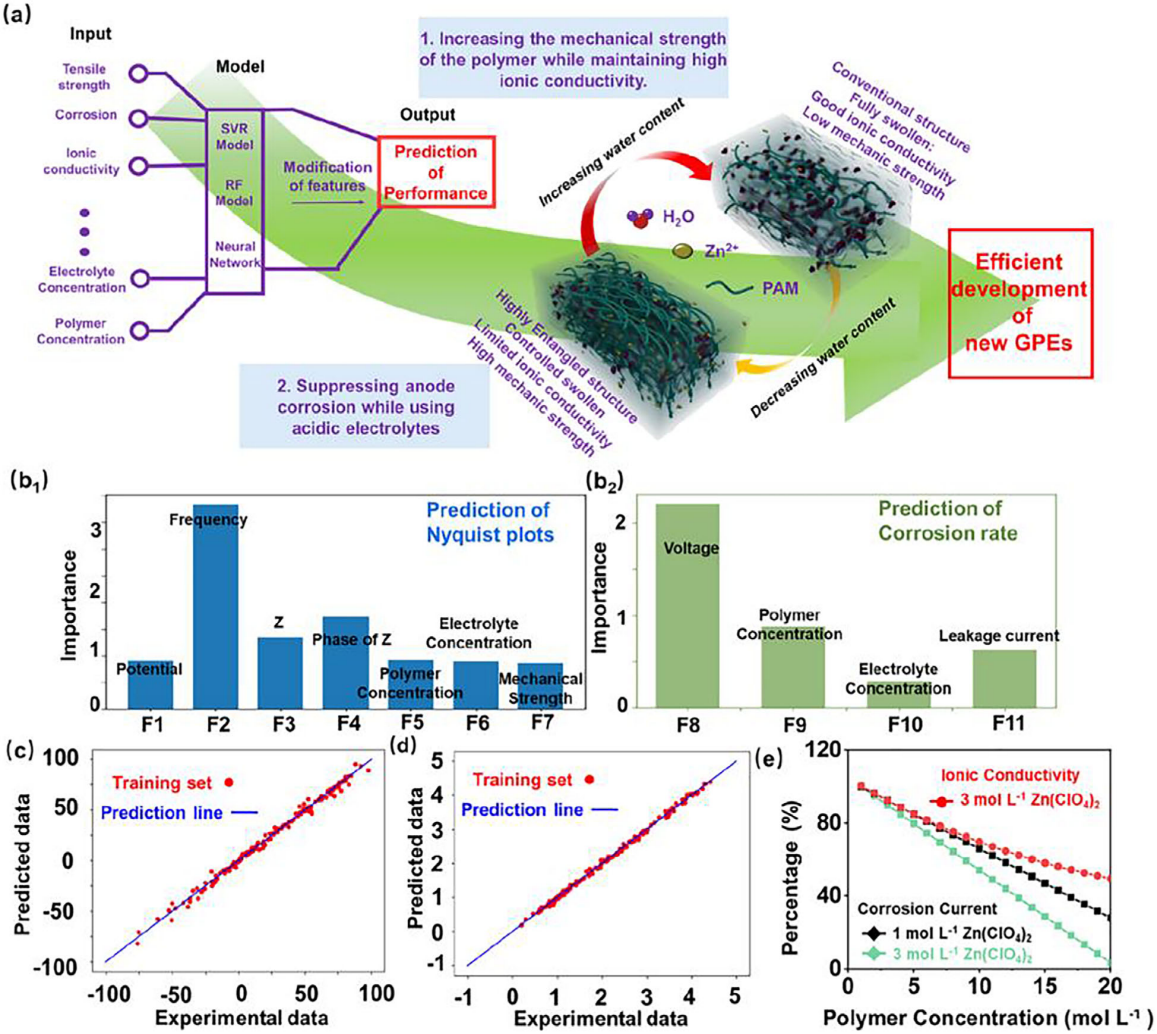
Machine‐learning‐guided design of gel polymer electrolytes (GPEs) for aqueous Zn‐metal batteries. (a) Schematic workflow illustrating the ML‐assisted design of polyacrylamide (PAM)‐based GPEs with tunable swelling and entanglement density. (b1, b2) Feature‐importance analysis for the prediction of AC impedance and corrosion‐related behavior, respectively. (c, d) Parity plots comparing experimentally measured and ML‐predicted values for corrosion rate and AC impedance, respectively. (e) Model‐predicted relationship between PAM concentration and ionic conductivity/corrosion current, illustrating the composition–property relationship used for experimental formulation selection; reproduced with permission [154], 2024, ACS Publications.

### 8.2 AI/ML‐Based Polymer and Electrolyte Screening

The chemical and architectural diversity of polymer electrolytes makes exhaustive experimental screening impractical. ML‐based screening therefore provides a systematic means of narrowing a very large candidate space to formulations that are most likely to satisfy application‐specific requirements. Such screening should be formulated as a multi‐objective problem rather than as optimization of a single property. For example, polymer‐host chemistry, counterion identity, molecular weight, and crosslinking density can alter ionic mobility, mechanical modulus, and thermal behavior in coupled ways [158]. Because polymer electrolytes frequently exhibit trade‐offs between ion transport and mechanical rigidity, data‐driven models are particularly valuable for identifying composition and processing windows that provide an acceptable balance among conductivity, mechanical integrity, electrochemical stability, and intelligent functionality. A central bottleneck in electrolyte discovery is not generating candidate polymers but efficiently narrowing an intractably large chemical space to a small subset suitable for synthesis. Tran et al. [159]. formalize this as a four‐stage, application‐driven screening protocol: (1) defining property‐based screening criteria tied to the target application, (2) constructing a candidate search space, (3) using trained ML models to predict relevant properties across that space, and (4) selecting and experimentally validating the candidates that satisfy the criteria, with failed or successful results feeding back into the training data in an iterative “active learning” loop. This closed‐loop structure, rather than any single algorithm, is what distinguishes ML‐assisted screening from other computational studies. The screening‐criteria step is illustrative of how this approach can be adapted across applications. For polymer dielectrics, criteria were defined around simultaneous thresholds for bandgap and dielectric constant, later extended to include glass transition temperature once high‐temperature stability became a design constraint. For instance, Gurnani et al. [160]. introduced polyVERSE, an AI‐driven framework that generates candidate polymers from commercially available monomers using established polymerization reaction templates, then screens them with multitask graph neural networks trained to predict glass transition temperature, bandgap, and dielectric constant. Applying this approach to ring‐opening metathesis polymerization (ROMP) chemistries, they screened over 26 000 candidate structures and identified PONB‐2Me5Cl, a previously unknown polynorbornene dielectric that achieved an energy density of 8.3 J cm^−^^3^ at 200°C, roughly 11 times higher than any commercially available polymer dielectric at that temperature. The same framework was extended to polyimides, yielding additional high‐performing, water/ethanol‐soluble candidates synthesizable from affordable starting materials. Their methodology is depicted in Figure 10a–c. A comparable logic applies directly to intelligent polymer electrolytes for zinc‐based systems, where candidate screening would similarly need to weigh ionic conductivity against mechanical strength, electrochemical stability window, and functional criteria such as antifreezing or self‐healing capacity, rather than optimizing any single property in isolation.

**FIGURE 10:**
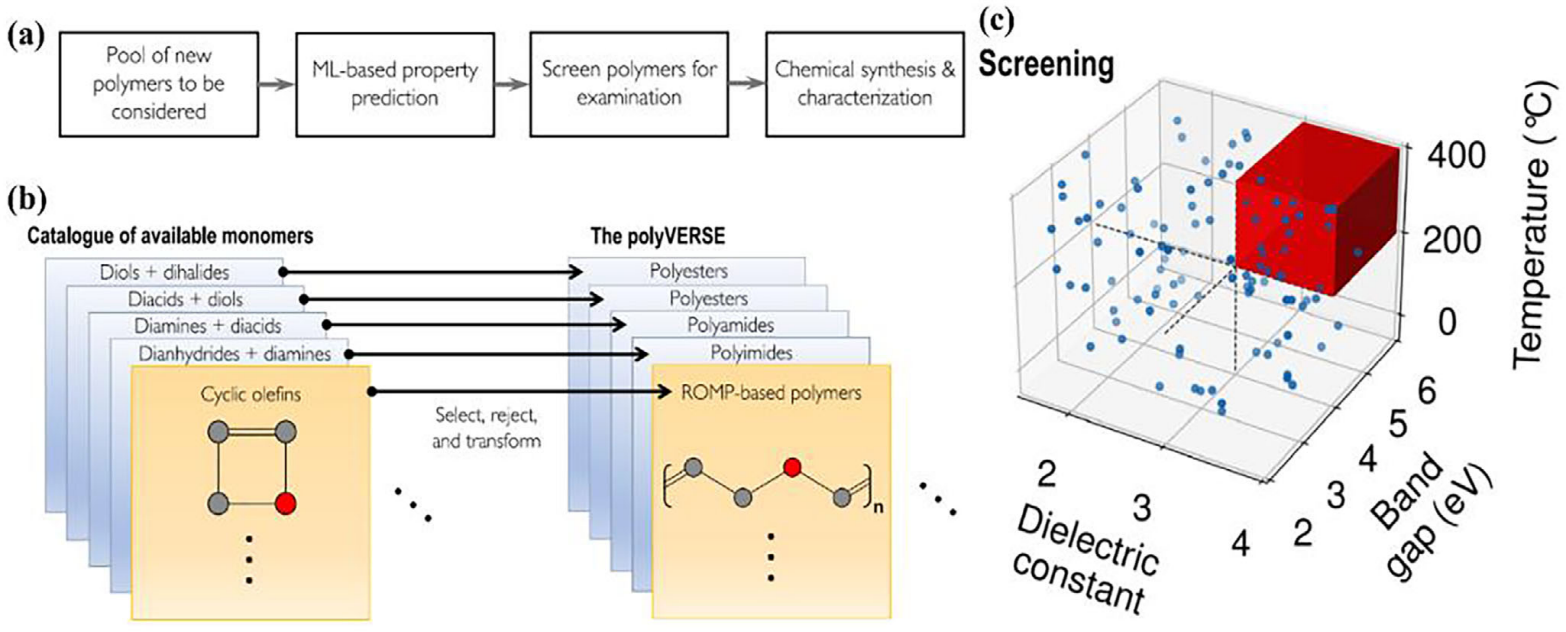
AI‐assisted design of polymers. (a) Four‐step design approach, (b) selection, rejection, and transformation of available monomers, and (c) screening performed using application‐specific filters [159].

### 8.3 DFT and AIMD for Molecular‐Level Understanding

Quantum‐mechanical methods such as density functional theory (DFT) provide high chemical accuracy but scale unfavorably with system size, with computational cost increasing approximately as the cube of the number of electrons or basis functions for many conventional implementations. This constraint generally limits routine DFT calculations to relatively small molecular or condensed‐phase models, which are insufficient to represent the long‐chain conformations, heterogeneous solvation environments, and extended network structures characteristic of realistic polymer electrolytes. Ab initio molecular dynamics (AIMD) inherits the same electronic‐structure cost because forces are recalculated from first principles at every time step, restricting simulations to comparatively small systems and short timescales. Classical molecular dynamics (cMD) circumvents this limitation by replacing explicit electronic‐structure calculations with parameterized interatomic force fields, thereby enabling substantially larger systems and longer trajectories at the expense of some chemical accuracy. Force fields can be broadly classified as general‐purpose models, which offer wide chemical coverage but may provide limited quantitative accuracy for a specific electrolyte, and system‐specific models, which can achieve higher fidelity for targeted chemistries but typically exhibit reduced transferability [161].

Earlier MD studies of Li^+^‐conducting polymer electrolytes have demonstrated how polymer architecture, ion dissociation, and chain structure influence ionic mobility. Although these studies provide useful methodological foundations, their direct applicability to aqueous Zn^2+^ systems are limited because Zn^2+^ exhibits different coordination chemistry, stronger electrostatic interactions, and coupled solvation/interfacial effects. Hua et al. used molecular dynamics simulations (Figure 11a,b) to investigate how anion chemistry and polymer main‐chain structure govern ionic conductivity in single‐Li^+^‐conducting solid polymer electrolytes (SLIC‐SPEs) dispersed in a PEO matrix. Although the study focused on Li‐based rather than Zn‐based electrolytes, its methodology illustrates how molecular simulation can separate the contributions of ion dissociation and segmental mobility to macroscopic conductivity. The authors first calibrated a force‐field charge‐scaling factor (n_s_ = 0.78) against experimentally measured conductivities of several lithium salts and then used an electric‐field drift method to evaluate a parameterized polymer architecture containing an anion intermediate group, an anion end group, and a variable‐length main chain. The simulations indicated that anion‐group chemistry primarily affected conductivity through the degree of salt dissociation, whereas main‐chain length and anion spacing influenced both dissociation and ion mobility, with mobility emerging as the stronger design lever for high‐conductivity polyanion architectures [161]. Wang et al. subsequently combined MD simulation with structural modeling to elucidate how maltose and a zwitterionic polymer backbone reshape Zn^2+^ transport in a crowded hydrogel electrolyte [162]. As summarized in Figure 11c,d, maltose bridges neighboring polymer chains through hydrogen‐bond interactions, generating confined domains that concentrate ions near dissociated charged sites on the polymer backbone. Radial‐distribution functions and coordination‐number analysis (Figure 11e,f) showed that Zn^2+^ in the liquid electrolyte is coordinated by approximately 4.34 H_2_O and 1.27 ClO_4_^−^, whereas in the crowded zwitterionic hydrogel electrolyte (CZHE), sulfonate groups directly participate in the Zn^2+^ solvation shell, reducing the corresponding H2O and ClO_4_^−^ coordination numbers to 3.94 and 1.10 and lowering the fraction of contact ion pairs from 42.5% to 24.4%. Mean‐square‐displacement profiles under an applied electric field (Figure 11g) further showed faster Zn^2+^ migration in the CZHE than in the liquid electrolyte. These simulation results provide a molecular‐level explanation for the high ionic conductivity of the CZHE (33.1 mS cm^−^^1^ at 25°C) and its stable operation down to −60°C.

**FIGURE 11:**
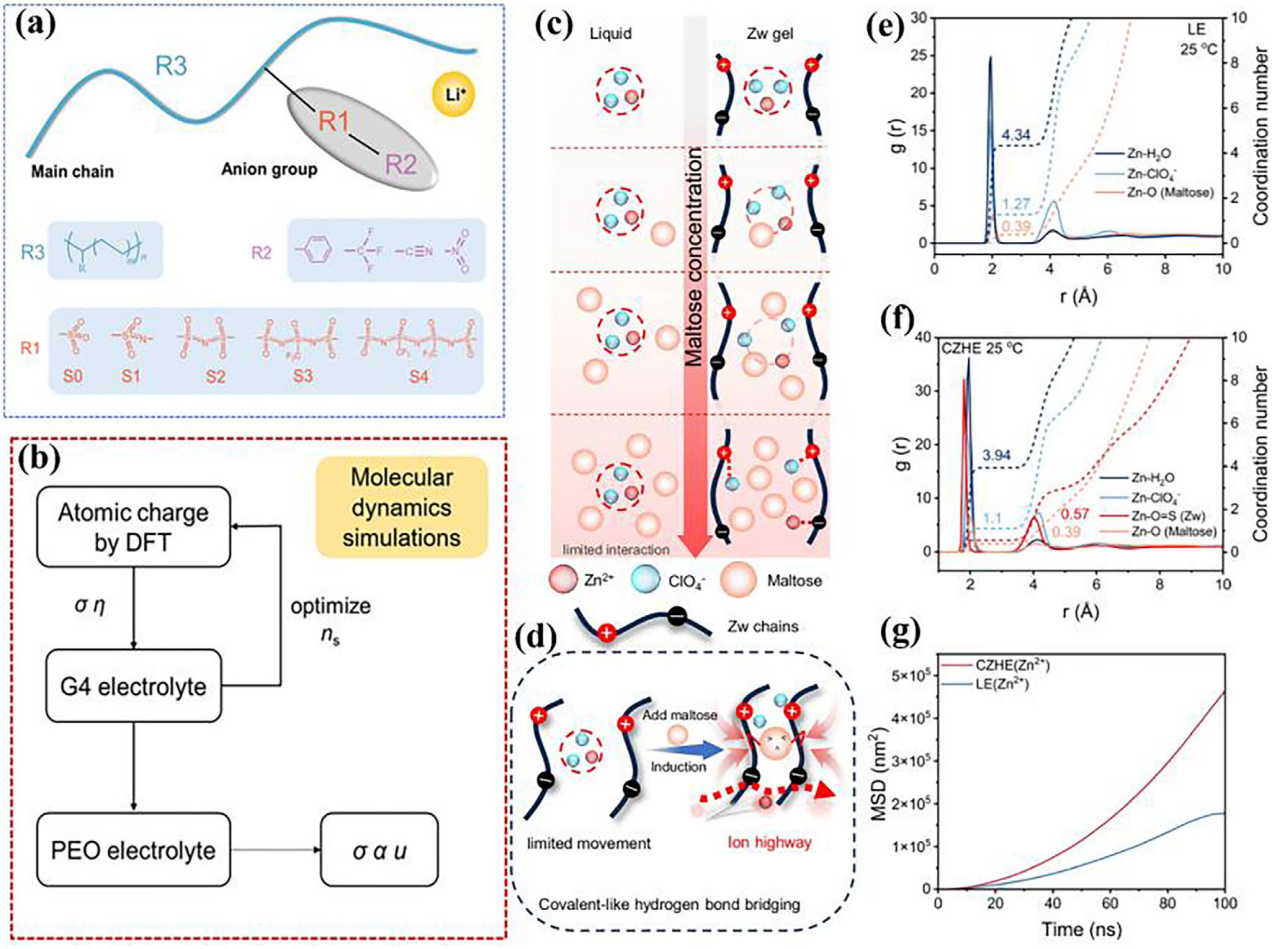
Representative applications of molecular dynamics (MD) simulation in polymer electrolyte design. (a) Modular structure of a sulfonimide‐based single‐lithium‐ion‐conducting polymer electrolyte (main chain R3, anion intermediate group R1, end group R2). (b) Workflow for calibrating the MD force field against experimental conductivity, dissociation degree, and mobility; reproduced with permission, [161] 2023, RSC. (c, d) Schematic of ion–polymer interactions in a zwitterionic (Zw) hydrogel electrolyte with increasing maltose concentration, and the resulting “ion highway” mechanism. (e, f) Radial distribution functions and coordination numbers for Zn^2+^ in the liquid electrolyte (LE) and crowded zwitterionic hydrogel electrolyte (CZHE) at 25°C. (g) Mean square displacement (MSD) of Zn^2+^ over time under an applied electric field for LE and CZHE [162].

### 8.4 Computational Bottlenecks and Strategies for Experimental Translation

Despite the increasing application of AI/ML, DFT, AIMD, and classical molecular dynamics to polymer‐electrolyte design, several limitations continue to restrict their direct translation into experimentally realizable materials. Among these limitations, dataset scarcity and inadequate chemical diversity are particularly important for data‐driven approaches because the available experimental datasets for polymer electrolytes are generally small, heterogeneous, and generated under different measurement conditions. Consequently, increasing dataset size alone is insufficient; the development of standardized datasets containing polymer composition, molecular descriptors, salt concentration, solvent or water content, crosslinking density, processing conditions, ionic conductivity, Zn^2+^ transference number, mechanical properties, electrochemical stability, and cycling performance measured under comparable conditions is required. Rather than treating these computational approaches independently, an integrated workflow can narrow the gap between prediction and experiment. In such a workflow, DFT and AIMD can first identify relevant molecular interactions and descriptors; classical molecular dynamics can subsequently evaluate ion coordination, diffusion, and transport in larger polymer networks; and uncertainty‐aware ML can use both experimental and simulation‐derived descriptors to prioritize experimentally accessible compositions. Active‐learning or closed‐loop experimental workflows can then iteratively select the most informative compositions for synthesis and testing, allowing the model to be updated using new experimental data. Importantly, computational screening should incorporate practical constraints such as polymer processability, precursor availability, crosslinking conditions, mechanical integrity, electrolyte loading, environmental stability, and compatibility with Zn electrodes rather than optimizing ionic conductivity alone. Such a multiscale and experimentally constrained strategy can progressively reduce the disconnect between predicted molecular properties and practical zinc‐battery performance.

## 9 Commercialization Challenges

### 9.1 Technical Challenges

Despite progress in intelligent polymer electrolytes for zinc‐based energy storage devices, several technical challenges must be addressed before large‐scale commercialization. The primary concern is balancing ionic conductivity, Zn^2+^ transport, mechanical robustness, flexibility, interfacial stability, and long‐term chemical stability. Polymer electrolytes can exhibit excellent laboratory‐scale performance, but their properties may change substantially with temperature, humidity, electrolyte composition, and operating conditions. In particular, Zn^2+^ coordination with polymer functional groups can improve ion regulation but may also reduce ion mobility when excessively strong interactions are established. Suppression of dendritic Zn growth, parasitic hydrogen evolution, corrosion, and cathode–electrolyte side reactions remain critical for achieving long cycle life. Intelligent polymer electrolytes incorporating self‐healing, stimuli‐responsive, antifreezing, or adaptive functionalities introduce additional complexity in synthesis, processing, and quality control. Maintaining uniform electrolyte thickness, crosslink density, additive distribution, and interfacial contact over large electrode areas is considerably more challenging than laboratory‐scale fabrication. Furthermore, the reproducibility of polymer networks and their electrochemical properties must be established across multiple batches. Standardized testing protocols for ionic conductivity, Zn^2+^ transference number, mechanical properties, temperature tolerance, and cycling durability are also needed. Integration with high‐loading electrodes and practical pouch or cylindrical cells remains insufficiently demonstrated. Therefore, commercialization requires a transition from material‐level optimization toward device‐level engineering, manufacturing consistency, safety validation, and long‐term reliability.

### 9.2 Economic Challenges

The economic viability of intelligent polymer electrolytes depends not only on the cost of individual components but also on precursor availability, synthesis complexity, processing energy, scalability, and manufacturing yield. Zinc‐based energy storage systems benefit from the relatively low cost and abundance of zinc; however, sophisticated polymer electrolytes containing specialty monomers, functional polymers, nanomaterials, ionic additives, DES components, and advanced crosslinkers can substantially increase material costs. Multi‐step synthesis involving purification, controlled polymerization, solvent exchange, post‐treatment, or incorporation of functional additives may also be difficult to translate into continuous industrial manufacturing. In particular, high‐performance intelligent electrolytes based on nanofillers, self‐healing polymers, or stimuli‐responsive molecular components may require expensive precursors or tightly controlled processing conditions. The economic advantage of zinc‐based systems can therefore be diminished if electrolyte manufacturing becomes significantly more expensive than conventional aqueous electrolytes. Another important consideration is electrolyte loading and manufacturing yield: excessive electrolyte uptake, long curing times, or batch‐to‐batch variation can increase production costs. Commercialization also requires compatibility with existing electrode coating, separator, cell assembly, and formation processes, minimizing the need for entirely new manufacturing infrastructure. Life‐cycle cost should include replacement frequency, cycle life, safety, maintenance, and end‐of‐life processing rather than relying solely on initial material cost. Consequently, future research should prioritize low‐cost and scalable monomers, bio‐derived polymers, abundant DES components, solvent‐minimized processing, rapid crosslinking, and roll‐to‐roll compatible fabrication. Demonstrating that intelligent polymer electrolytes can provide substantially longer lifetime, improved safety, or wider operating temperatures at a competitive cost will be essential for establishing their commercial value.

### 9.3 Environmental Challenges

Environmental sustainability is an important consideration in the commercialization of intelligent polymer electrolytes for zinc‐based energy storage devices. Although zinc‐based batteries offer advantages associated with zinc abundance, relatively favorable safety, and the potential for aqueous operation, the overall environmental impact depends strongly on the composition and manufacturing of the polymer electrolyte. Conventional synthetic polymers, fluorinated components, organic solvents, crosslinkers, and certain nanomaterials may generate environmental burdens during synthesis, use, and disposal. Similarly, the incorporation of DESs or plasticizers does not automatically guarantee environmental benignity because their toxicity, biodegradability, persistence, and life‐cycle impacts depend on the individual components and their concentrations. The environmental fate of polymer electrolytes after battery disposal therefore requires systematic evaluation. Another challenge is the difficulty of separating crosslinked polymer electrolytes from electrodes and other cell components during recycling. Intelligent functionalities may further increase compositional complexity and complicate material recovery. Consequently, future designs should emphasize bio‐derived polymers, non‐toxic crosslinkers, recyclable or biodegradable electrolyte components, water‐based processing, and earth‐abundant additives while maintaining electrochemical performance. Life‐cycle assessment should be incorporated from the early development stage to evaluate raw‐material extraction, polymer synthesis, cell manufacturing, operational durability, and end‐of‐life treatment. Designing polymer electrolytes for disassembly, recovery, and recycling could substantially reduce their environmental footprint. Ultimately, environmentally responsible commercialization requires moving beyond the simple replacement of liquid electrolytes with polymeric systems toward a life‐cycle‐oriented design strategy in which sustainability, safety, performance, and recyclability are simultaneously considered. The principal commercialization barriers identified in this review are compared in Table 4, with emphasis on the validation and design responses required for translation beyond laboratory‐scale demonstrations.

**TABLE 4:** Commercialization‐oriented comparison of the major challenges facing intelligent polymer electrolytes for zinc‐based energy‐storage devices.

Challenge domain	Article‐identified bottleneck	Why it matters for scale‐up	Priority response / validation
Technical	Ionic conductivity balance. Zn^2+^ transport efficiency. Mechanical and chemical stability. Temperature‐dependent reproducibility.	Non‐uniform electrolyte thickness. Inconsistent crosslink density. Poor interfacial contact. Increased cell variability.	Standardized electrochemical testing. Mechanical stability assessment. Multi‐batch reproducibility. Practical cell validation.
Economic	Expensive specialty monomers. Costly functional additives. Complex synthesis procedures. Low manufacturing yield.	Increased production costs. Reduced cost competitiveness. Limited manufacturing compatibility.	Low‐cost precursor selection. Bio‐derived material utilization. Rapid crosslinking processes. Continuous manufacturing compatibility.
Environmental	Toxic chemical components. Persistent fluorinated materials. Solvent‐intensive processing Difficult polymer recycling.	Increased environmental burden. Complex waste management. Limited material recovery.	Bio‐derived polymer utilization. Non‐toxic crosslinker selection. Water‐based processing. Life‐cycle assessment.

## 10 Future Perspectives

The rapid development of zinc‐based energy‐storage technologies is increasing the demand for polymer electrolytes that combine high ionic conductivity with interfacial stability, mechanical durability, and environmental adaptability. Future systems should move beyond passive ion‐conducting matrices toward multifunctional and adaptive platforms capable of regulating local electrochemical conditions in real time. Promising directions include AI‐guided materials discovery, single‐ion‐conducting architectures, self‐healing and antifreezing networks, bio‐derived polymers, printable electrolytes, and integrated electrolyte–electrode structures. The practical impact of these concepts will ultimately depend on whether laboratory‐scale functionality can be translated into reproducible, scalable, and manufacturable devices. The principal research directions discussed below are summarized schematically in Figure 12.

**FIGURE 12:**
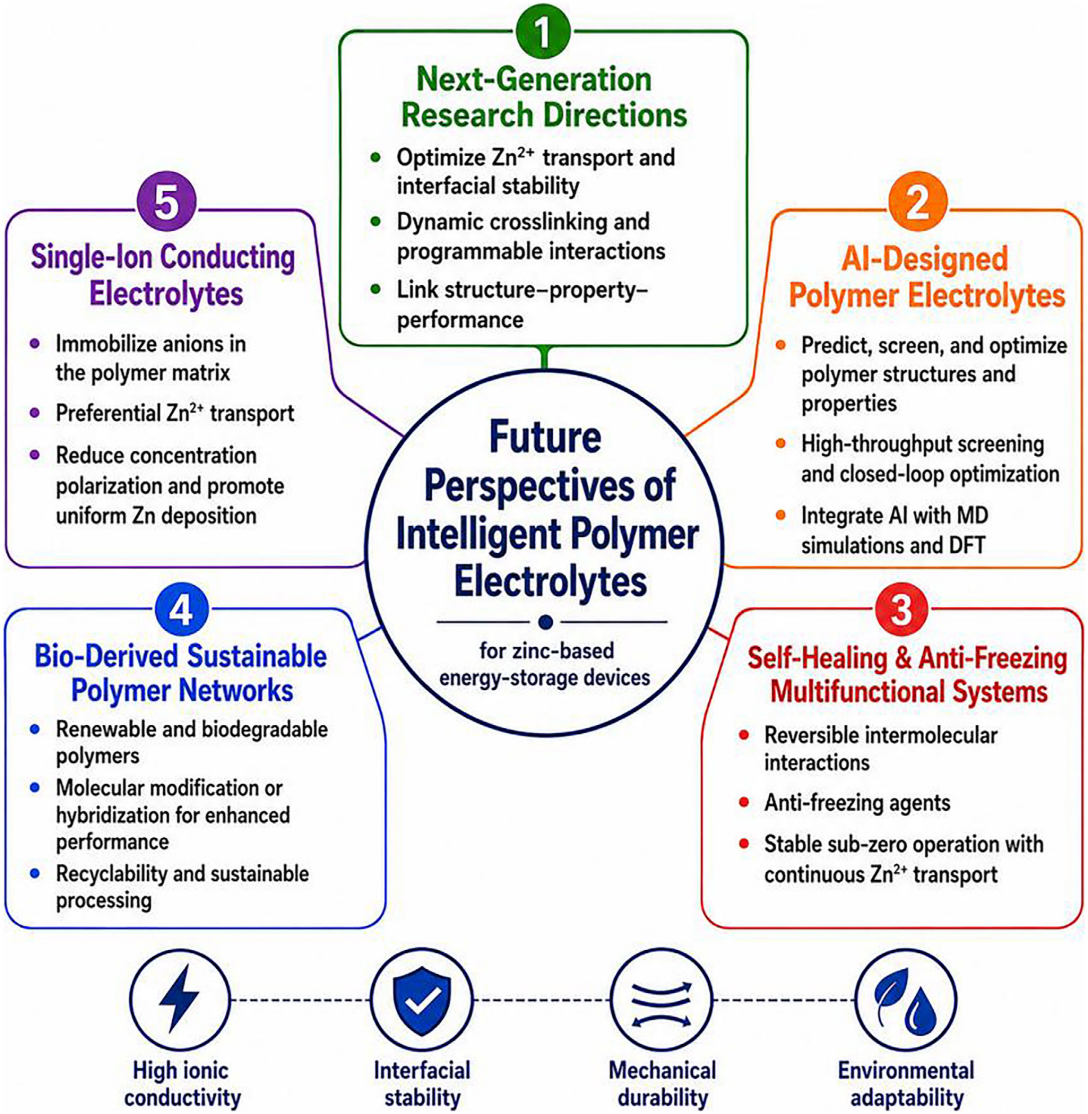
Generalized future research directions for intelligent polymer electrolytes in zinc‐based energy‐storage devices.

### 10.1 Next‐Generation Research Directions

Future research on intelligent polymer electrolytes should move beyond maximizing any single descriptor, particularly ionic conductivity or symmetric‐cell lifetime, and instead establish coupled structure–property–performance relationships that account for ion transport, water regulation, interfacial stability, mechanical durability, and environmental adaptability. The central design challenge is to manage the trade‐offs among these properties: stronger polymer–ion interactions may improve selectivity yet slow transport; denser networks may suppress dendrite penetration yet increase resistance; and stronger water confinement may improve thermal tolerance while reducing low‐temperature kinetics. Polymer networks should therefore be engineered with dynamic crosslinks, programmable molecular interactions, and hierarchical transport pathways that remain effective under variations in current density, temperature, humidity, and mechanical deformation. Progress will also require closer integration of operando characterization, molecular simulation, and data‐driven modeling to identify the molecular origins of transport and degradation. The long‐term objective is not simply a multifunctional electrolyte, but an adaptive electrolyte whose functions remain effective under practically relevant operating conditions and scalable manufacturing constraints.

### 10.2 AI‐Designed Polymer Electrolytes

Artificial intelligence (AI) and machine learning (ML) are expected to accelerate the discovery and optimization of intelligent polymer electrolytes by reducing reliance on sequential trial‐and‐error experimentation. Their value, however, will depend more on data quality, descriptor relevance, and experimental consistency than on model complexity alone. Data‐driven models can relate polymer backbone chemistry, crosslinking strategy, electrolyte composition, additives, and processing conditions to target properties such as ionic conductivity, Zn^2+^ transference number, mechanical strength, electrochemical stability, and cycling durability. When coupled with high‐throughput synthesis and automated electrochemical testing, these methods could enable closed‐loop optimization in which model predictions guide experiments and new measurements continuously improve the model. Integration of ML with molecular dynamics and DFT can further provide physically informed descriptors for Zn^2+^ coordination, polymer segmental dynamics, water activity, and interfacial reactions. Standardized datasets, explicit reporting of uncertainty, and prospective experimental validation will therefore be essential before AI‐guided electrolyte design can become reliably predictive rather than primarily retrospective. Such closed‐loop, experimentally autonomous optimization should therefore be regarded as a prospective development pathway, rather than as a generally demonstrated capability of the polymer‐electrolyte systems reviewed here.

### 10.3 Self‐Healing and Antifreezing Multifunctional Systems

Future intelligent polymer electrolytes should integrate multiple functions within a single platform while retaining robust electrochemical performance. Importantly, multifunctionality should not be equated with simply accumulating more functions; each added feature must remain electrochemically useful under the same operating conditions. Self‐healing and antifreezing capabilities are particularly important for flexible devices exposed to repeated mechanical damage and subzero environments. Reversible hydrogen bonding, metal–ligand coordination, ionic interactions, host–guest chemistry, and dynamic covalent bonds can restore network connectivity and ion‐transport pathways after damage, whereas polyols, deep eutectic solvents, hydrated salts, and polymer–water interactions can suppress ice crystallization. The key challenge is to combine these mechanisms without excessive crosslink density, viscosity, or water confinement that compromises Zn^2+^ mobility. Multifunctional systems should therefore be evaluated through coupled mechanical, transport, and full‐cell measurements under realistic temperature and deformation cycles rather than through isolated demonstrations of each function.

### 10.4 Bio‐Derived Sustainable Polymer Networks

Sustainability should be treated as a primary design criterion for next‐generation intelligent polymer electrolytes rather than as a secondary advantage. Bio‐derived polymers such as cellulose, SA, CMC, chitosan, starch, gelatin, lignin, and carrageenan provide renewable and potentially biodegradable alternatives to petroleum‐derived matrices. Their abundant hydroxyl, carboxyl, and amino groups offer multiple sites for Zn^2+^ coordination, hydrogen bonding, and dynamic crosslinking, enabling the construction of mechanically robust ion‐conducting networks. Future work should focus on molecular modification, copolymerization, and hybridization strategies that overcome excessive swelling, insufficient mechanical strength, and limited long‐term chemical stability. Sustainable plasticizers, natural deep eutectic solvents, and bio‐based nanofillers may further improve water retention, ionic conductivity, and interfacial stability. Importantly, claims of sustainability should be supported by scalable synthesis, recyclability or biodegradability assessments, and life‐cycle considerations rather than by feedstock origin alone.

### 10.5 Single‐ion Conducting Electrolytes

Single‐ion‐conducting polymer electrolytes offer a promising route to reducing concentration polarization and increasing the fraction of current carried by Zn^2+^ in zinc‐based energy‐storage devices. In these systems, anionic groups are immobilized on or within the polymer framework, leaving Zn^2+^ as the dominant mobile charge carrier. A higher Zn^2+^ transference number can homogenize cation flux, reduce concentration gradients, and promote more uniform Zn nucleation and deposition. Future designs should therefore explore immobilized sulfonate, phosphate, carboxylate, borate, or related anionic motifs while carefully balancing ion dissociation strength and cation mobility. Combining single‐ion conduction with dynamic crosslinking, self‐healing behavior, and temperature‐adaptive networks may provide additional functionality, but such integration must avoid overly strong Zn^2+^ binding that would suppress conductivity.

### 10.6 Printable Electrolytes for Wearable Devices

The rapid expansion of wearable electronics and soft robotics has created increasing demand for printable polymer electrolytes capable of fabricating lightweight, flexible, and mechanically robust zinc‐based energy storage devices. Future intelligent polymer electrolytes should possess suitable rheological properties for advanced additive manufacturing techniques, including inkjet printing, screen printing, aerosol jet printing, direct ink writing, and three‐dimensional printing. The electrolyte inks must simultaneously exhibit excellent printability, rapid solidification, strong substrate adhesion, and high ionic conductivity without sacrificing flexibility or electrochemical stability. Tailoring polymer viscosity, crosslinking kinetics, and solvent evaporation behavior will be essential for producing defect‐free electrolyte patterns with controlled thickness and uniform ion‐conducting pathways. Furthermore, printable electrolytes should maintain stable performance under repeated bending, stretching, twisting, and compression while remaining compatible with textile fibers, elastomers, and flexible current collectors. Such manufacturing strategies will facilitate scalable production of personalized, lightweight, and conformable zinc‐based energy storage systems for next‐generation wearable and portable electronics.

### 10.7 Multifunctional Electrolyte‐Electrode Integration

Future zinc‐based energy‐storage devices should transition from individually optimized components toward fully integrated multifunctional architectures in which the electrolyte and electrodes operate synergistically. Intelligent polymer electrolytes should be engineered to actively interact with electrode surfaces through controlled chemical bonding, interfacial coordination, and dynamic molecular interactions, thereby minimizing contact resistance and facilitating efficient Zn^2+^ transport across electrode–electrolyte interfaces. Functional groups incorporated within polymer networks can regulate zinc nucleation, stabilize catalytic active sites, and suppress undesirable side reactions, resulting in improved electrochemical reversibility and prolonged cycling stability. Simultaneously, the integration of conductive nanomaterials, redox‐active polymers, catalytic nanoparticles, and porous electrode frameworks into continuous monolithic structures can shorten ion diffusion pathways while enhancing mechanical integrity. Future studies should explore interface‐engineered architectures using operando characterization and multiscale simulations to understand interfacial evolution during battery operation. Such integrated systems are expected to deliver higher energy density, lower internal resistance, improved durability, and simplified device fabrication for advanced zinc‐based energy storage technologies.

### 10.8 Autonomous Self‐Adaptive Energy Storage Systems

The next generation of intelligent polymer electrolytes should enable autonomous energy‐storage systems capable of continuously adapting to changing electrochemical and environmental conditions without external intervention. These adaptive electrolytes should dynamically regulate Zn^2+^ transport, ionic conductivity, water content, and interfacial stability in response to variations in temperature, humidity, mechanical deformation, current density, or state of charge. Dynamic polymer networks incorporating reversible covalent bonds, supramolecular interactions, or stimuli‐responsive functional groups can continuously reorganize their molecular structure to preserve ion‐conducting pathways and mechanical integrity during long‐term operation. Integration with embedded microsensors and intelligent battery management systems could further enable real‐time monitoring of electrolyte health, ion transport behavior, and degradation processes, allowing predictive maintenance and self‐optimization of device performance. Coupling adaptive polymer chemistry with artificial intelligence algorithms may ultimately create self‐learning electrolyte systems capable of autonomously optimizing operational parameters, thereby significantly improving the safety, efficiency, durability, and reliability of future zinc‐based energy storage devices. At present, fully integrated sensing–feedback–autonomous regulation remains a prospective direction rather than a broadly experimentally demonstrated capability of intelligent polymer electrolytes. Future generations of these materials and devices may enable autonomous energy‐storage systems capable of continuously adapting to changing electrochemical and environmental conditions without external intervention.

### 10.9 Commercial‐Scale Flexible Zinc Battery Technologies

Commercial deployment of flexible zinc‐based energy‐storage devices will require intelligent polymer electrolytes that combine electrochemical performance with scalable processing, reproducibility, and long‐term reliability. Future formulations should be compatible with roll‐to‐roll coating, extrusion, continuous casting, and large‐area printing while maintaining uniform thickness, composition, and crosslink density. Beyond material‐level tests, a minimum practical validation set should include controlled electrolyte thickness and loading, high cathode mass loading or areal capacity, limited Zn excess, long‐term full‐cell cycling, and retention of function under relevant temperature and deformation histories. Standardized testing of ionic conductivity, adhesion, bending and puncture resistance, environmental stability, and device lifetime will be essential for comparing technologies across laboratories. In parallel, recyclable polymer matrices, environmentally benign synthesis routes, and abundant raw materials should be prioritized to control both manufacturing cost and environmental impact. Close collaboration among materials scientists, electrochemists, process engineers, and computational researchers will be necessary to translate laboratory‐scale intelligent polymer electrolytes into commercially viable flexible zinc‐based energy‐storage devices.

## 11 Summary

Intelligent polymer electrolytes represent a fundamental shift from passive ion‐conducting media toward materials that actively regulate ion transport, interfacial chemistry, water activity, and mechanical integrity in zinc‐based energy‐storage devices. The literature reviewed here can be understood as a progression from active physicochemical regulation, to adaptive response, and ultimately toward autonomous or data‐guided electrolyte operation. Across ZIBs, ZABs, and ZIHCs, the central challenge is therefore not to maximize the number of incorporated functions, but to ensure that each function addresses a defined failure mode without creating a new transport, mechanical, manufacturing, or sustainability penalty. Hydrogels, organohydrogels, composite polymer electrolytes, and solid‐state polymer electrolytes provide complementary routes to this objective, while self‐healing, antifreezing, adhesive, stretchable, and stimuli‐responsive designs extend the electrolyte from a transport medium to an adaptive electrochemical component. Bio‐derived polymers and AI‐, ML‐, MD‐, and DFT‐assisted design further broaden the available design space. Future progress should prioritize mechanism‐based multifunctional integration, standardized and practically relevant testing, scalable fabrication, sustainability, and rigorous full‐cell validation; in this context, electrolyte “intelligence” should ultimately be judged by demonstrated regulation under operating conditions rather than by functionality claims alone.

## Author Contributions

**Sanath Kumar**: Conceptualization, Data curation, Investigation, Writing – original draft. **Jhansirani Kesavan**: Data curation, Investigation, Writing – original draft. **Muhammad Asim**: Data curation, Investigation, Writing – original draft. **Atul Verma**: Data curation, Investigation, Writing – original draft. **Yen‐Pei Fu**: Conceptualization, Funding acquisition, Investigation, Project administration, Supervision, Writing – review and editing.

## Conflicts of Interest

The authors declare no conflicts of interest.

## Data Availability

The data that support the findings of this study are available from the corresponding author upon reasonable request.
